# Glucose transporters in brain in health and disease

**DOI:** 10.1007/s00424-020-02441-x

**Published:** 2020-08-13

**Authors:** Hermann Koepsell

**Affiliations:** Institute for Anatomy and Cell Biology, University of Würzburg, Koellikerstr 6, 97070 Würzburg, Germany

**Keywords:** Glucose transporter, Brain, GLUT1, GLUT2, GLUT3, GLUT4, SGLT1, Diabetes, Parkinson’s disease, Stroke, Traumatic brain injury, GLUT1 deficiency syndrome

## Abstract

Energy demand of neurons in brain that is covered by glucose supply from the blood is ensured by glucose transporters in capillaries and brain cells. In brain, the facilitative diffusion glucose transporters GLUT1-6 and GLUT8, and the Na^+^-d-glucose cotransporters SGLT1 are expressed. The glucose transporters mediate uptake of d-glucose across the blood-brain barrier and delivery of d-glucose to astrocytes and neurons. They are critically involved in regulatory adaptations to varying energy demands in response to differing neuronal activities and glucose supply. In this review, a comprehensive overview about verified and proposed roles of cerebral glucose transporters during health and diseases is presented. Our current knowledge is mainly based on experiments performed in rodents. First, the functional properties of human glucose transporters expressed in brain and their cerebral locations are described. Thereafter, proposed physiological functions of GLUT1, GLUT2, GLUT3, GLUT4, and SGLT1 for energy supply to neurons, glucose sensing, central regulation of glucohomeostasis, and feeding behavior are compiled, and their roles in learning and memory formation are discussed. In addition, diseases are described in which functional changes of cerebral glucose transporters are relevant. These are GLUT1 deficiency syndrome (GLUT1-SD), diabetes mellitus, Alzheimer’s disease (AD), stroke, and traumatic brain injury (TBI). GLUT1-SD is caused by defect mutations in GLUT1. Diabetes and AD are associated with changed expression of glucose transporters in brain, and transporter-related energy deficiency of neurons may contribute to pathogenesis of AD. Stroke and TBI are associated with changes of glucose transporter expression that influence clinical outcome.

## Introduction

Glucose transporters in brain play pivotal roles in various brain functions in health and disease. The high energy demand of neurons is mainly covered by d-glucose supply with the blood that is accomplished by glucose transporters in capillaries and brain cells. In addition to energy supply during neurotransmission, cerebral glucose transporters are critically involved in sensing of glucose concentrations in blood, cerebrospinal fluid (CSF), and brain interstitium promoting central nervous and whole-body regulatory processes. Glucose transport across the blood-brain barrier (BBB) and across plasma membranes of neurons and glial cells is precisely regulated. This is necessary because energy demand changes in response to brain activity. In addition, the delivery of d-glucose to brain is not constant and changes due to alterations in blood glucose concentration and in blood pressure. Various diseases are associated with, aggravated by, and/or caused by impairment of central nervous supply with oxygen and/or glucose. Examples include diabetes mellitus, Parkinson’s disease (PD), stroke, and traumatic brain injury (TBI). In brain, facilitative diffusion transporters belonging to the *SLC2* family including the transporters GLUT1, GLUT2, GLUT3, and GLUT4, and Na^+^-d-glucose cotransporters belonging to the *SLC5* family including SGLT1 have been detected. In this review, an attempt is made to provide a comprehensible overview of the current knowledge about functions of glucose transporters in brain. First, the functional properties and substrate selectivities of human glucose transporters expressed in brain are reviewed and the locations of glucose transporters in brain are described. Because only few data about cerebral locations of glucose transporters in human are available, the described locations are mostly derived from studies in rodents. In the second chapter, the roles of glucose transporters in central nervous regulation of glucose homeostasis are discussed. This includes the sites of glucose sensing in brain and the central regulation of insulin and glucagon secretion. Like in the previous and the following chapter, most of the reported insights are derived from studies with rodents. The third chapter deals with various types of regulations of glucose transporters in response to energy demands. This includes short-term regulations of glucose transporters in different cerebral cells and regions during learning and exercise. In the fourth and fifth chapters, associations of diabetes and Alzheimer’s disease (AD) with changed expression and functions of glucose transporters in brain and with intellectual impairments are reported. Two hypotheses concerning the pathogenesis of AD that complement each other are outlined. In addition, data are reported suggesting that downregulation of GLUT1 and GLUT3 leading to a decrease of the d-glucose concentration in neurons represents an early event during the pathogenesis of AD. In the next chapter, GLUT1 deficiency syndrome (GLUT1-DS) is described. In the last two chapters, the changes of cerebral glucose transporters during stroke and traumatic brain injury (TBI) are reported and the impact of glucose transporters on clinical outcome of these devastating events is discussed. A detailed list of references is provided to allow in-depth reading.

## Locations and functional properties of glucose transporters expressed in brain

### Overview

**Fig. 1:**
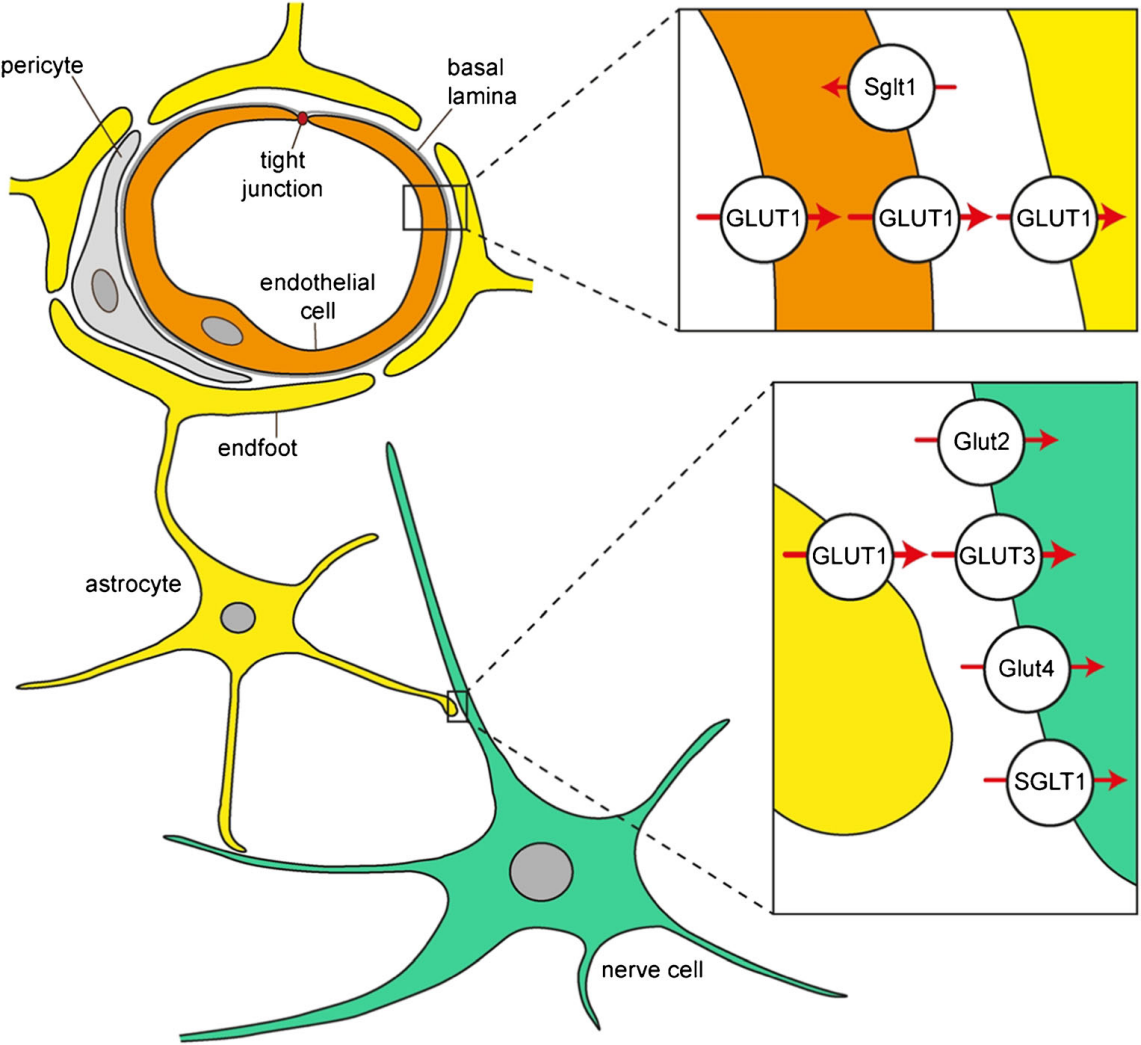
Schematic depiction of a brain capillary, an associated astrocyte, and an interacting neuron with the most relevant glucose transporters. Capillary endothelial cells that are connected by tight junctions form the blood-brain barrier. In the insets, glucose transporters are depicted that mediate d-glucose transport across the indicated membranes. The main direction of d-glucose translocation is shown by red arrows. Transporters are denoted by capital letters when their locations were described in humans and rodents. Lowercase letters were used when the transporter locations were only described in rodents

**Fig. 2:**
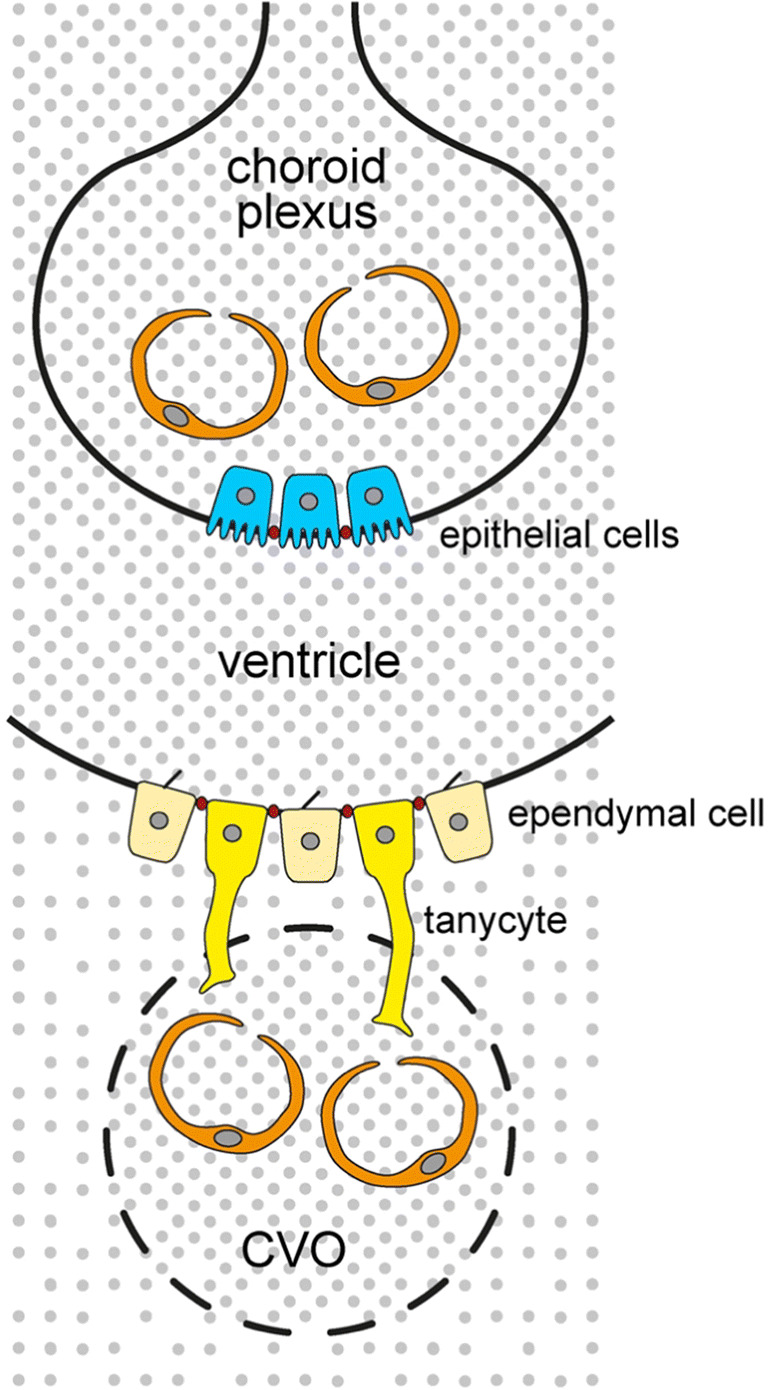
Barriers between blood and CSF and between brain interstitium and CSF containing glucose transporters. A barrier between blood in the choroid plexus and CSF in brain ventricles is formed by epithelial cells covering the choroid plexus. Tanycytes form a barrier between blood in CVOs and CSF in brain ventricles. A barrier between brain interstitium and CSF is formed by ependymal cells including tanycytes that line brain ventricular walls. Tight junctions are indicated in red. Different concentrations of d-glucose in the compartments are indicated by the density of gray dots

About 20% of ingested d-glucose is consumed by human brain [278]. To enter brain interstitium or brain ventricles, d-glucose must pass the blood-brain barrier (BBB) (Fig. 1), the barrier between choroid plexus and cerebrospinal fluid (CSF) in brain ventricles, the barrier between brain interstitium and brain ventricles, or the barrier between circumventricular organs (CVOs) and brain ventricles (Fig. 2) [7, 333]. The BBB is formed by endothelial cells that are connected through tight junctions (Fig. 1) [44]. The barrier between blood and CSF in the choroid plexus is formed by tight junction-connected epithelial cells (Fig. 2) [44]. The barrier between brain interstitium and CSF is formed by ependymal cells lining brain ventricles that are also connected by tight junctions, and the barrier between blood and CSF at CVOs is formed by tanycytes (Fig. 2) [333]. CVOs contain leaky capillaries. They include the subfornical organ, the area postrema, the vascular organ of the lamina terminalis, and the median eminence (ME) [106]. Because hydrophilic compounds like d-glucose cannot transverse tight junctions and need transporters to cross plasma membranes, glucose transporters are expressed in luminal and abluminal plasma membrane of capillary endothelial cells, plasma membranes of epithelial cells covering the choroid plexus, and plasma membranes of ependymal cells and tanycytes. To allow uptake of d-glucose into brain cells, glucose transporters are also expressed in neurons, astrocytes, oligodendroglial cells, and microglial cells.

The glucose transporters expressed in brain belong to *SLC2* transporter family containing GLUT-type facilitated diffusion transporters and the *SLC5* family containing SGLT-type Na^+^-d-glucose cotransporters (Table 1). To fulfill different requirements such as optimal transport efficacies at different glucose concentrations and physiological demands, different types of glucose transporters are expressed in different brain areas and cells (Tables 2 and 3). Collaborative functions of glucose transporters in the BBB, glial cells, and neurons are involved in maintenance of energy supply to neurons.

**Table 1:** Apparent *K*_m_ values [mM] of *trans-*zero d-glucose uptake by human glucose transporters that are expressed in brain

Transporter	d-Glucose	d-Galactose	d-Fructose	2-Deoxy-glucose	3-*O*-Methyl-glucose	Reference
GLUT1	0.7–3.2	tr.	no tr.	6.9	1.4	[49, 362, 438, 439, 442]
GLUT2	17–20	86	67	11, 17	17	[49, 72, 144, 193, 407]
GLUT3	~ 1.5	8.5	no tr.	1.4, 1.8	10.6	[49, 72, 143, 144, 400]
GLUT4	12.6	tr.	no tr.	4.6	4.3	[49, 297, 439, 442]
GLUT5	not t. for tr.	not te.	6	tr.	not te.	[50, 198, 203]
GLUT6	tr.	not te.	not te.	tr.	not te.	[53, 103]
GLUT8	tr.	i., not te. for tr.	i., not te. for tr.	2.4	not te.	[104, 178]
SGLT1	0.5	1	no tr.	> 100	> 100	[446]
SGLT2	5	> 100	> 100.	not te.	not te.	[446]

*tr.* transport, *no tr.* no transport, *i.* inhibition, *not te. for tr.* not tested for transport, *not te.* not tested

**Table 2:** Expression of glucose transporters in cerebral neurons and glial cells

Transporter	Neurons	Astrocytes	Oligodendrocytes	Microglial cells	Reference
GLUT1/Glut1	+ (ro.)	+++ (hu., ro.) hyth.	+ (ro.)	+ (ro.)	[83, 245, 247, 319, 433, 458]
Glut2	+ (ro.) bst., cort., th., hyth., hip	+ (ro.)	+ (ro.)		[12, 13, 83, 230, 294]
GLUT3/Glut3	+++ (hu., ro.) bst., mb., cereb., cort., hyth., hip.	+ (ro.)		+ (ro.)	[11, 40, 63, 64, 83, 185, 252, 283, 291]
Glut4	+ (ro.) bst., mb., cereb., bg., cort., olb., hyth., hip.,	+ (ro.)		+ (ro.)	[64, 108, 109, 209, 231, 363, 420]
GLUT5/Glut5	+ (ro.) cereb., cort., hyth., nopt.			+ (hu., ro.)	[130, 212, 247, 321]
GLUT8/Glut8	+ (ro.) mb., cereb., cort., hyth., hip., olb.			+ (ro.)	[1, 79, 341, 363, 433]
SGLT1/Sglt1	+ (hu., ro.) cereb., cort., hyth., hip.	+ (ro.)			[22, 110, 118, 202, 305, 330, 422, 460]

*hu.* human, *ro.* rodent, *bst*. brainstem, *mb*. midbrain, *bg.* basal ganglia, *cort*. cerebral cortex, *olb*. olfactory bulb, *cereb*. cerebellum, *hip.* hippocampus, *th.* thalamus, *hyth*. hypothalmus, *nopt*. nuclei of the optical tract

**Table 3:** Expression of glucose transporters in capillary endothelial cells, choroidal epithelial cells, ependymal cells, and tanycytes

Transporter	Capillary endothelial cells	Choroidal epithelial cells	Ependymal cells	Tanycytes	Reference
GLUT1/Glut1	+++ (hu., ro) lu., ablu.	+ (ro.)	+ (ro.)	+ (ro.)	[39, 40, 75, 77, 102, 107, 120, 131, 132, 134, 155, 218, 245, 247, 380, 385, 426, 427]
Glut2			+ (ro.)	+ (ro.)	[24, 83, 132, 245, 258, 294]
GLUT3/Glut3	+ (hu., ro.)				[3, 113, 135, 137, 252]
Glut4	+ (ro.)	+ (ro.)	+ (ro.)		[108, 209, 245, 266, 421]
GLUT5/Glut5	+ (hu.)	+ (hu., ro.)	+ (hu., ro.)	+ (ro.)	[212, 253, 406]
Glut6			+ (ro.)	+ (ro.)	[258, 391]
GLUT8/Glut8	+ (ro.)	+ (ro.)	+ (hu., ro.)		[288, 292, 319]
Sglt1	+ (ro.)				[110, 224]
Sglt2	+ (ro.)				[113]

*hu.* human, *ro.* rodent, *lu.* luminal membrane, *alu*. abluminal membrane

Translocation of d-glucose across the BBB is mainly mediated by the high-affinity transporter GLUT1 that is highly expressed in the luminal and abluminal membranes of the endothelial cells (Fig. 1). In small brain vessels, additional glucose transporters were observed such as Glut3 and Glut4 and the Na^+^-d-glucose cotransporter Sglt1 (Table 3). These transporters may serve specific local functions. The driving force for facilitative diffusion of d-glucose across the BBB by the GLUT transporters is provided by the concentration gradient between d-glucose in blood and brain interstitium. Between meals, the d-glucose concentration in the blood is 4–6 mM whereas the d-glucose concentration in brain interstitium is only 1–2 mM [319]. The glucose concentration gradient between blood and brain interstitium is supposed to be generated and sustained by uptake of d-glucose into astrocytes and neurons, and metabolic degradation of d-glucose in these cells. SGLT1/Sglt1-mediated uptake from brain interstitium into the capillary endothelial cells may contribute (Fig. 1).

**Fig. 3:**
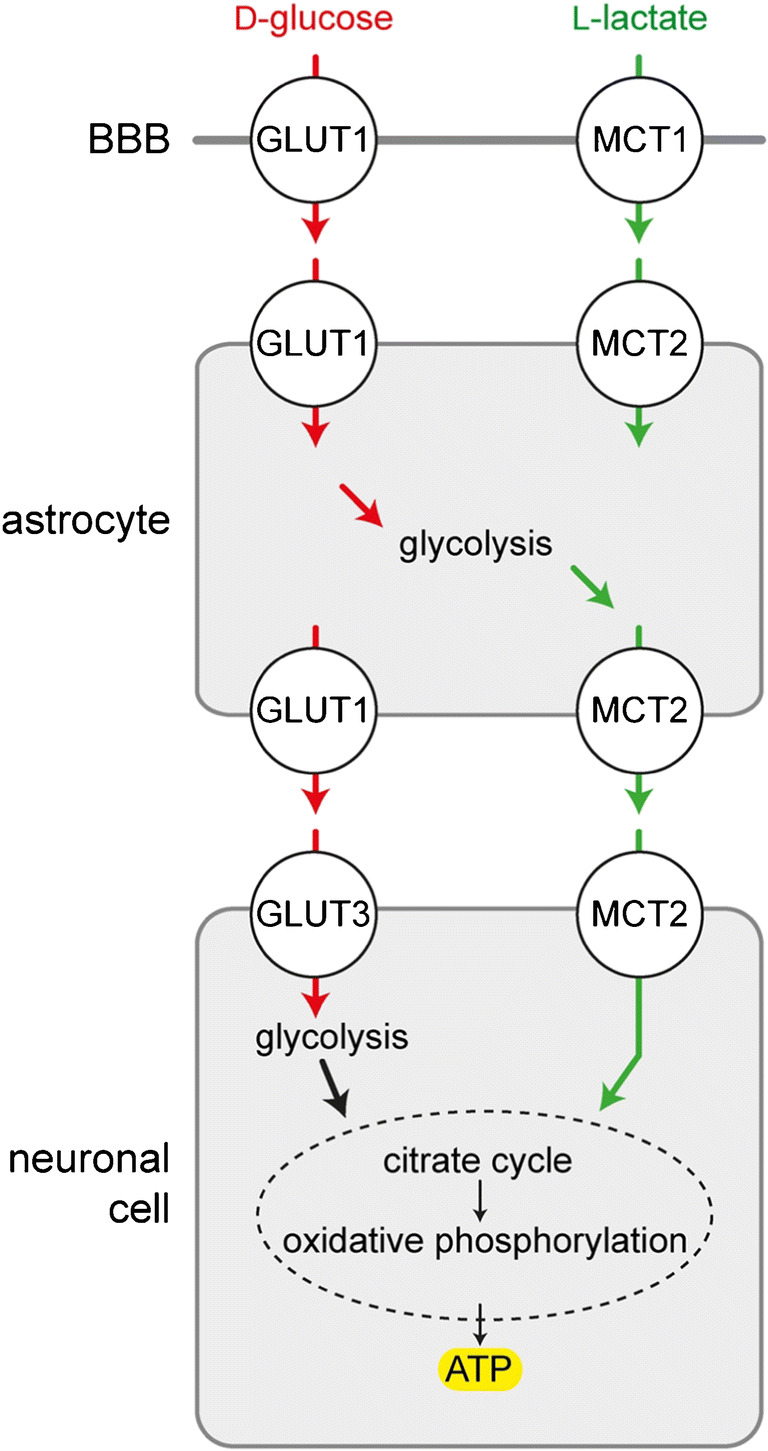
Role of astrocytes for transfer of d-glucose and l-lactate from blood to nerve cells supplying energy in the form of ATP. During hypoglycemia or nutrition through a ketogenic diet, energy may be derived from l-lactate supplied with the blood. l-Lactate may be also generated by astrocytes and contribute to neuronal energy supply under normal conditions as proposed by the astrocyte-lactate-neuron shuttle hypothesis. MCT1 monocarboxylate transporter 1, MCT2 monocarboxylate transporter 2

Similar to endothelial cells in the BBB, the high-affinity GLUT1 transporter is highly expressed in dendritic end-feet of astrocytes that enwrap brain capillaries and are connected by permeable gap junctions [7] (Fig. 1). In addition, expression of low-affinity Glut2, Glut3, and insulin-dependent Glut4 in astrocytes has been observed (Table 2). The biggest part of d-glucose leaving the capillary endothelial cells is supposed to enter the end-feet of astrocytes where it may be metabolized to l-lactate or leave astrocyte processes close to neurons. A smaller fraction of d-glucose leaving the endothelial cells is supposed to enter the interstitial space directly. d-Glucose uptake into neurons is mainly mediated by GLUT3, a high-affinity glucose transporter that operates with high efficacy (Fig. 1, Table 1). Additional transporters may participate in d-glucose uptake into neurons that are critical for special functions in specific brain areas and/or under specific physiological or pathophysiological conditions (Table 2). For example, neuronal expression of Glut2 and Glut4 has been described in hypothalamic nuclei where these transporters are involved in central regulations of glucohomeostasis, food intake, and/or energy balance. SGLT1 which is ubiquitously expressed in neurons may be important for glucose uptake under hypoglycemic and hypoxemic conditions.

d-Glucose taken up by neurons enters glycolysis and is further metabolized by oxidative phosphorylation (Fig. 3). However, energy delivery to neurons may be also accomplished by uptake of l-lactate that is supplied by astocytes or directly by the blood during ketogenic metabolism (Fig. 3) [394]. l-Lactate leaves the astrocytes via the monocarboxylate transporter MCT2 and enters neurons via MCT2 [31, 138, 337]. The role of d-glucose uptake into astrocytes followed by the astrocyte-lactate-neuron shuttle versus direct uptake of d-glucose into neurons under normal physiological conditions is controversially discussed [28, 250, 251, 324]. However, there is an agreement that in case of insufficient supply with d-glucose or upon nutrition with ketogenic diet, l-lactate in the blood may become essential for central nervous energy supply. l-Lactate can enter and leave brain capillaries via MCT1 in the luminal and abluminal membrane of the endothelial cells [229, 319].

In the following parts of this chapter, the basic transport characteristics of the human glucose transporters expressed in brain are reviewed. In addition, the cerebral locations of glucose transporters determined in humans and/or rodents are reported and their presumed cerebral functions are compiled.

### GLUT1

Human GLUT1 transports d-glucose, d-galactose, d-glucosamine, and the glucose analogs 2-deoxy-d-glucose (2DOG) and 3-*O*-methyl-d-glucose (3OMG) (Table 1). For uptake of d-glucose and 3OMG by GLUT1 measured in the absence of initial intracellular substrate (*trans-*zero uptake), *K*_m_ values between 0.7 and 3.2 were determined. For *trans-*zero uptake of 2DOG a *K*_m_ value of 6.9 mM was measured. GLUT1 also accepts dehydroascorbic acid as substrate [1, 204, 362, 424]. In addition, evidence was provided that human GLUT1 facilitates uptake of water and trivalent arsenicals via a translocation pathway different to d-glucose [124, 182, 192, 235].

In various species, GLUT1/Glut1 is abundantly expressed in endothelial cells of the BBB exhibiting different expression levels in different brain regions (Table 3) [40, 77, 155, 426, 427]. In brain of humans and primates, capillaries with high and low expression of GLUT1 were distinguished [76–78]. GLUT1 in small brain vessels isolated from pig and dog was highly glycosylated and appeared in SDS polyacrylamide gels as 55 kDa polypeptide like in human erythrocytes [94, 134, 201, 380]. In the BBB, the 55 kDa GLUT1 polypeptide was localized to the luminal membrane, the cytosol, and the abluminal membrane of capillary endothelial cells. Studies on isolated luminal and abluminal membranes of endothelial cells from bovine brain vessels revealed that GLUT1 in the luminal membrane was highly phosphorylated whereas GLUT1 in the abluminal membrane showed minor phosphorylation [93]. Employing different antibodies for electronmicroscopic immune detection of GLUT1/Glut1 in different species, diverging results concerning the abundance of GLUT1/Glut1 in the luminal versus the abluminal membrane of capillary endothelial cells were reported [75, 120, 134, 380, 385]. However, comparing d-glucose equilibrium exchange in vesicles of luminal and abluminal membranes of capillary endothelial cells from bovine brain and binding of cytochalasin B to isolated luminal and abluminal membranes, transport and binding was about twofold higher in the luminal compared to the abluminal membrane [380]. This result was confirmed by proteomic analysis [217]. In human brain vessels, endothelial cells with high and low expression of GLUT1 were distinguished by immunogold electron microscopy [74, 78]. The 55 kDa isoform of Glut1 was also localized to the basolateral membrane of epithelial cells in the choroid plexus of rat, mouse, and rabbit [39, 102, 107, 155]. Abundant expression of non-glycosylated GLUT1/Glut1 with an apparent molecular mass of 45 kDa was observed in astrocyte of human, monkey, and rat where it was located to end-feet surrounding capillaries, dendrites close to neurons, and astrocyte cell bodies (Fig. 1) [228, 282, 458]. Glut1-mediated glucose uptake into cultured astrocytes was stimulated by glutamate suggesting that astrocytes participate in metabolic upregulation during neuronal activity [331]. In rodents, expression of Glut1 was also observed in oligodendrocytes, microglia, neurons, ependymal cells, and tanycytes [131, 155, 218, 245, 247, 319, 433, 458].

The abundant expression of GLUT1/Glut1 in capillary endothelial cells and end-feet of astrocytes indicates that this transporter is of major relevance for the transfer of d-glucose across the BBB and into astrocytes.

### GLUT2

Human GLUT2 is a low-affinity glucose transporter with apparent *K*_m_ values for *trans-*zero uptake of 17–20 mM for d-glucose, 86 mM for d-galactose, and 67 mM for d-fructose (Table 1). For uptake of 2DOG and 3OMG, similar *K*_m_ values as for d-glucose uptake were reported. GLUT2 also functions as a glucose receptor that triggers glucose-dependent upregulation of GLUT2 expression via its large intracellular loop [152, 390]. After overexpression of the large intracellular loop of rat Glut2 in mice, d-glucose-induced upregulation of Glut2 expression was blunted and food uptake was increased. In this transgenic mouse, d-glucose-induced activation of c-Fos in the hypothalamic arcuate nucleus (ARH) was defective and the abundance of orexin mRNA in hypothalamus was increased.

GLUT2 is abundantly expressed in hepatocytes but also expressed in pancreatic β cells and brain. In pancreatic β cells, GLUT2 serves as sensor for blood glucose in combination with the pancreatic glucokinase (GK) and an ATP-dependent K^+^ channel [399]. In brain of rodents, expression of Glut2 was detected in thalamic nuclei, in hypothalamic nuclei including the ARH, in nuclei of the brain stem including the nucleus of the tractus solitarius and the vagal motor nucleus, and in hippocampus [12, 24, 230]. In addition, Glut2 was observed in CVOs [258, 294]. Glut2 is expressed in neurons, astrocytes, oligodendrocytes, ependymal cells, and tanycytes (Tables 2 and 3) [12, 13, 24, 83, 132, 230, 245, 258, 294].

Glut2 is supposed to be involved in regulation of food and glucose intake and in the central nervous regulation of glucose homeostasis. When cerebral expression of Glut2 in rats was reduced by injection of antisense oligonucleotides into the third brain ventricle, food intake was decreased [430]. In addition, the increase of food intake observed after injection of 2DOG into the third ventricle was blunted when the cerebral expression of Glut2 had been reduced by antisense technology. Similar effects of cerebral removal of Glut2 on food intake were observed in mice. In Glut2 knockout mice in which expression of Glut2 in pancreatic β cells was rescued by expression of rat Glut1, food intake was smaller than in wildtype mice [20]. Moreover, the effects of intracerebroventricular (i.c.v.) injection of d-glucose or 2DOG to decrease or increase food intake, respectively, were blunted in the knockout mice. In the knockout mice, also glucagon secretion in response to glucodeprivation induced by i.c.v. injection of 2DOG was blunted [259]. Glucagon secretion was restored when Glut2 expression in glial cells was recovered by transgenesis. A study with two Canadien populations suggests that also in human, GLUT2 is involved in central nervous control of d-glucose ingestion [114]. A single nucleotide variation in GLUT2 leading to one amino acid exchange was correlated with an increased glucose uptake independently of age and T2DM.

Impact of GLUT2/Glut2 in brain on glucose-dependent central nervous regulation of insulin secretion and glucagon secretion was suggested by two studies. In one study performed with rats, the expression of Glut2 in the ARH was decreased by bilateral injection of antisense oligonucleotides, and insulin secretion was analyzed after injection of a small amount of d-glucose into a carotic artery [232]. The injected glucose did not increase the d-glucose concentration in the blood. Whereas the intracranial d-glucose bolus stimulated insulin secretion in control rats, no stimulation of insulin secretion was observed in rats that had been treated with Glut2 antisense oligonucleotides. In another study, an impact of Glut2 in brain on central nervous stimulation of glucagon secretion during d-glucose depletion was demonstrated in glut2 knockout in which the glut2 loss in pancreatic β cells was rescued [259]. In wildtype mice, glucagon secretion was increased after intraventricular application of 2DOG mimicking glucoprivation; however, no central nervous stimulation of glucagon secretion was observed in the Glut2 knockout mice. Of note, evidence was provided that this effect was due to removal of Glut2 in astrocytes rather than to removal of Glut2 in neurons. This demonstrates a pivotal metabolic coupling between astrocytes and neurons.

Recent data suggest that Glut2 in tanycytes of the ME containing leaky capillaries is involved in translocation of d-glucose from the interstitium into the third ventricle [258]. In the presence of high d-glucose concentrations in the blood, the glucose concentration in third ventricle increased correspondingly whereas the d-glucose concentration in brain tissue with functional BBBs only increased slightly. The elevated d-glucose concentration in the third ventricle observed in response to an increase of blood glucose was blunted when the expression of Glut2 and Glut6 in tanycytes of the ME had been reduced by siRNA technology [258].

Experiments performed with Zebrafish expressing a GLUT2 orthologoue in hindbrain in which the GLUT2 orthologoue was removed or rescued suggested that GLUT2 also plays an important role during brain development [256].

### GLUT3

Human GLUT3 mediates *trans*-zero uptake of d-glucose and 2DOG with similar, relatively low *K*_m_ values around 1.5 mM (Table 1). This value is in the same range as the *K*_m_ value for d-glucose uptake by human GLUT1. Human GLUT3 does not accept d-fructose as substrate but transports d-galactose and 3OMG with 5–8 times higher *K*_m_ values than d-glucose (Table 1). Comparing the turnover numbers for d-glucose transport by Glut3 in rat cerebellar neurons and by human GLUT1 in erythrocytes, an about fivefold higher turnover number was obtained for Glut3 [248, 382]. Provided this difference is not due to species differences, the data suggest that GLUT3 transports glucose much more efficiently than GLUT1. Similar to human GLUT1, human GLUT3 increases transmembrane water permeability [402].

In situ hybridization and immunolocalization experiments performed in rodents, monkeys and humans indicate that GLUT3/Glut3 is ubiquitously expressed in brain. GLUT3/Glut3 was detected in the frontal and parietal cerebral cortex, hippocampus, gyrus pyriformis, corpus striatum, cerebellum, inferior colliculi, and brainstem [252, 263, 283, 291, 372, 455]. In brain, GLUT3/Glut3 is predominantly expressed in neurons. Neuronal expression was demonstrated by localization of GLUT3/Glut3 in various nuclei of the brain stem, in the substantia nigra, the granular cell layer and dentate nucleus of cerebellum, in brain cortex, hippocampus, and hypothalamus (Table 2) [11, 40, 63, 64, 83, 252, 283, 291]. In neurons, GLUT3/Glut3 was located in neurites, dentrites, and plasma membranes of the cell bodies [135, 228, 252, 382]. High expression was observed in pre- and postsynaptic nerve endings. In cultured granular neurons derived from rat cerebellum, a six- to tenfold higher abundance of Glut3 was observed compared to Glut1 [246]. Expression of GLUT3/Glut3 was also detected in brain microvessels where it was localized to endothelial cells [3, 113, 135, 137, 252]. Minor expression of Glut3 was detected in cultured astrocytes derived from rat [185]. Because GLUT3/Glut3 is ubiquitously and abundantly expressed in brain neurons, this transporter is supposed to serve housekeeping uptake of d-glucose into neurons.

### GLUT4

GLUT4/Glut4 is an insulin-sensitive glucose transporter that plays a key role in regulation of body glucose homeostasis. GLUT4/Glut4 is most abundantly expressed in adipose tissue, skeletal muscle, and heart. It is transferred from intracellular compartments into the plasma membrane in response to extracellular insulin [174]. After ingestion of glucose-rich food when blood glucose is increased and pancreatic insulin secretion is induced, accelerated insulin-mediated d-glucose uptake into adipocytes and muscle cells counterregulates the elevation of blood glucose [467]. This regulatory circuit is defective in T2DM in which pancreatic insulin secretion is impaired and the sensitivity of insulin receptors in fat and muscle cells is decreased. Human GLUT4 transports d-glucose, d-galactose, 2ODG, and 3OMG but does not accept d-fructose as substrate (Table 1). For *trans-*zero uptake of d-glucose by human GLUT4, an apparent *K*_m_ value of 12.6 mM was determined [442], whereas for *trans-*zero uptake of 2DOG, an apparent *K*_m_ value of 4.6 mM has been reported [49]. Similar to GLUT1 and GLUT3, GLUT4 accepts dehydroascorbic acid as substrate [350].

Employing in situ hybridization and immunohistochemistry in rodents, low-level expression of Glut4 was observed in motor nuclei of spinal cord, nuclei of medulla oblongata, cerebellar nuclei and Purkinje cell layer, basal ganglia, neocortex, olfactory bulb, hypothalamus, and hippocampus (Table 2) [64, 108, 109, 209, 231, 420]. Glut4 is mainly expressed in neurons where it is often coexpressed with Glut3 [11]. Here, Glut4-related immunoreactivity was predominantly observed in the somatodendritic portion; however, immunoreactivity was also detected in neurites [108, 209, 231, 363]. Glut4-related immunoreactivity in neuronal somata was mostly assigned to intracellular compartments [108]. In general, Glut4 protein and Glut4 mRNA showed similar differences in abundance between brain areas. However, in some locations, differences were observed between relative abundance of mRNA and protein indicating posttranscriptional regulation [43, 109]. Low abundant expression of Glut4 was also detected in endothelial cells of microvessels from rat brain [108, 266]. In rodents, Glut4 was also detected in epithelial cells of the choroid plexus and in ependymal cells of brain ventricles [209, 245, 421]. Of note, glut4 in neurons was often colocalized with the insulin receptor [150, 199, 420]. In cultivated neurons, insulin-induced incorporation of Glut4 from intracellular stores into the plasma membrane was demonstrated [30, 150].

GLUT4/Glut4 in brain is supposed to be involved in provision of metabolic energy for firing neurons, in insulin-dependent regulation of active neuronal circuits, and in central nervous regulation of whole-body glucose homeostasis. The increased energy demand in firing neurons is met by upregulation of ATP synthesis [338]. For generation of ATP by glycolysis and mitochondrial ATP synthesis, intracellular glucose is required. Evidence was provided that increased energy demand during sustained neuronal activation promotes the insertion of Glut4 into the axonal plasma membrane, and that the Glut4 insertion is under control of AMP activated protein kinase (AMPK) [16]. In motoric neurons, energy demand is acutely increased during exercise whereas energy demand in hippocampal neurons is increased in response to intellectual challenge or emotional stress.

Insulin plays important regulatory roles in brain where it interacts with the insulin receptor in neurons located in various brain areas including forebrain, hypothalamus, and hippocampus [81, 411]. Insulin may exhibit direct effects as well as d-glucose-mediated effects on neuronal activity [81, 215]. Insulin passes the BBB and the barriers between blood and CSF very slowly, and the concentration of insulin in CSF is one order of magnitude lower than in blood [81, 429]. Evidence was presented that insulin is synthesized by subpopulations of cortical and hippocampal neurons and by neuronal progenitor cells [81, 220]. Brain-derived insulin is supposed to provide local stimuli for rapid upregulation of GLUT4/Glut4 in neurons with high energy demand that may not be covered by GLUT3/Glut3-mediated glucose uptake [81, 112].

**Fig. 4:**
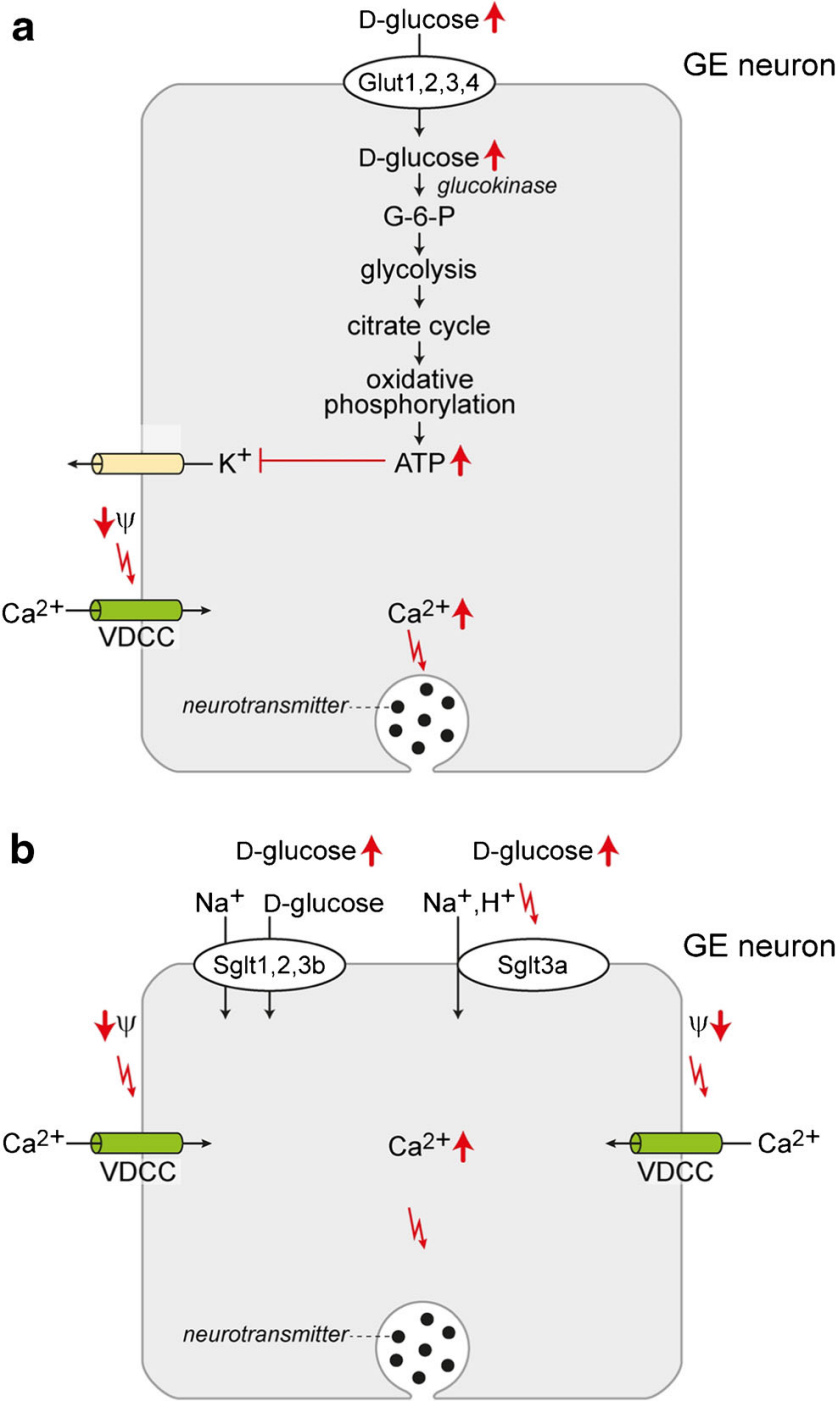
Involvement of glucose transporters and a glucose sensor in d-glucose sensing by neurons that are excitated by d-glucose (GE neurons). **a** A metabolism-dependent mechanism detected in rodents is shown. Increased d-glucose uptake at high extracellular glucose by a Glut transporter leads to an increase of intracellular glucose promoting ATP synthesis. Elevated intracellular ATP blocks an ATP-dependent K^+^ channel resulting in a decrease of the membrane potential. This promotes opening of the voltage-dependent Ca^2+^ channel VDCC. Increased intracellular Ca^2+^ induces the release of neurotransmitters. **b** A metabolism-independent mechanism observed in rodents is shown. Na^+^-d-glucose cotransport by Sglt1, Sglt2, or Sglt3b or binding of d-glucose to the glucose activated Na^+^/H^+^ ion channel Sglt3a leads to a depolarization of the plasma membrane and to an increase of Ca^2+^ uptake via VDCC. The increased intracellular Ca^2+^ concentration triggers the release of neurotransmitters. Ψ membrane potential

GLUT4/Glut4 is supposed to be also involved in hypothalamic regulation of food intake, energy expenditure, and whole-body glucohomeostasis [345, 346]. Increased or decreased concentrations of d-glucose in brain activate different neurons in hypothalamus that either decrease or increase endogeneous d-glucose production (EGP) in the liver. Hypoglycemic counterregulation that is crucial for insulin-treated diabetic patients involves central effects of insulin, sympathoadrenal stimulation, and increase of pancreatic glucagon secretion [41, 126, 299, 313]. The glucose-dependent activation of hypothalamic neurons may occur directly by d-glucose uptake into efferent neurons or indirectly by d-glucose-mediated activation of insulin secretion by interconnecting neurons and insulin-induced upregulation of GLUT4/Glut4 in efferent d-glucose-sensitive neurons. After removal of Glut4 in mouse brain, the glucose-dependent regulation of glucohomeostasis was blunted [346]. Data have been reported which suggest that Glut4 is involved in d-glucose sensing in hypothalamic nuclei [199]. In neurons of the dissociated ventromedial hypothalamic nucleus (VMH), d-glucose-sensitive neurons were identified by measuring d-glucose-induced effects on oscillations of intracellular Ca^2+^ concentrations. It was observed that more than 60% of neurons that were stimulated when extracellular d-glucose was either increased or decreased coexpressed Glut4 and the insulin receptor. In most d-glucose excitable neurons, also GK was expressed and d-glucose activation was abolished when GK was inhibited by alloxan. GK has a gate keeping function for d-glucose-induced increase of intracellular ATP (Fig. 4a).

Prolonged changes of d-glucose and insulin concentrations in brain and decreased insulin receptor sensitivity during diabetes may influence the expression and function of GLUT4/glut4 in brain. This may result in permanent alterations of plasticity of neuronal circuits. In cultivated human cells, the expression of GLUT2 was decreased and glucose-dependent incorporation of GLUT4 into the plasma membrane was decreased after chronic treatment with insulin [30]. In mice, the abundance of Glut4 in the hypothalamus was decreased when the insulin receptor in neurons had been removed [97].

### GLUT5

Human GLUT5 can be considered as selective transporter for d-fructose with the restriction that minor uptake of 2DOG has been described [50, 198, 203]. For *trans-*zero uptake of d-fructose by human GLUT5, an apparent *K*_m_ of 6 mM was determined (Table 1).

In addition to intestine, skeletal muscle, fat, testis, and spermatozoa, human GLUT5/Glut5 is expressed in brain [50, 203, 371]. In rodents, Glut5 has been localized to various brain regions including cerebral cortex, hippocampus, cerebellum, and nuclei of the brain stem [212, 308]. In human and rat, abundant expression of GLUT5/Glut5 was observed in microglial cells [247, 321]. In human, GLUT5 expression was also detected in microvascular endothelial cells [253] whereas in rodents, expression of Glut5 was observed in cerebellar Purkinje cells, nuclei of the optical tract, cortical and hypothalamic neurons, epithelial cells of the choroid plexus, ependymal cells, and tanycytes [130, 212, 253, 406]. Oxidative metabolism of d-fructose does not only occur in liver, kidney, and small intestine but also in brain. Accordingly, in rodents, considerable amounts of d-fructose injected into brain or applied to brain tissue sections were metabolized [164, 308]. After injection of [^14^C] d-fructose into rat brain and after incubation of isolated nerve terminals with [^14^C] d-fructose, ^14^C labeling of alanine, glutamate, aspartate, γ-aminobutyric acid (GABA), and glutamine was observed [164]. d-Fructose may enter oxidative metabolism directly employing ketohexokinase (KHK), triokinase, and aldolase or indirectly following conversion to d-glucose after phosphorylation by hexokinase. In brains of mice and/or rats, expression of KHK, aldolase, and hexokinase 1 was observed [164, 308]. Expression of KHK was demonstrated in Purkinje cells of mouse cerebellum [130]. Fructose may enter the brain via GLUT5/Glut5 in capillary endothelial cells, choroidal epithelial cells, ependymal cells, or tanycytes. In early experiments, no significant or minimal d-fructose uptake into brain was observed after injection of tracer amounts of radioactively labeled d-fructose into the carotic artery [304, 401]. This is not surprising because the concentration of d-fructose in the blood between meals is about three orders of magnitude lower than the concentration of d-glucose [312]. However, d-fructose oxidation in brain becomes relevant after ingestion of fructose-rich food, particularly in combination with different forms of fructose intolerance. Feeding of rats for 5 days with d-fructose resulted in an about twofold increase of Glut5 in hippocampus [377]. It was observed that the enzymatic activity of KHK in brain was threefold increased in mice that had been provided for 1 month with drinking water containing 40% d-fructose [308]. An enhanced metabolism of d-fructose in brain has been shown to induce the formation of advanced glycation endproducts that are associated with several brain pathologies including AD [121, 164]. Noteworthy, high d-fructose concentrations in diets induced a central neuronal insulin resistance and promoted memory impairment in animal models of dementia [56, 276].

### GLUT6

Human GLUT6, originally named GLUT9, may be considered as low-affinity d-glucose transporter because transport of 5 mM d-glucose was demonstrated after reconstitution into protoliposomes whereas no significant transport of 1 mM d-glucose was observed [103, 194]. Using endometrial tumor cells that overexpressed GLUT6, it was shown that GLUT6 also accepts 2DOG as substrate. In human and mouse, abundant expression of GLUT6/Glut6 mRNA was observed in brain and spleen [54, 103]. Expression of Glut6 mRNA was also detected in leukocytes, heart, and pancreas of humans and in macrophages of mice [58, 103, 244]. In mouse brain, Glut6 protein was demonstrated in the ME and the ARH and localized to ependymal cells and tanycytes [258, 391].

GLUT6/Glut6 is preferentially located in intracellular compartments including lysosomes and supposed to undergo insulin-independent endocytotic recycling [233, 244, 258]. After expression of hemagglutinin-epitope-tagged human GLUT6 in primary rat adipose cells, GLUT6 was nearly exclusively observed in intracellular compartments [233]. Similarly, Glut6-related immunoreactivity in tanycytes of the ME was mostly observed inside the cells [258]. GLUT6 and the structural closely related glucose transporter GLUT8 contain N-terminal dileucine motifs that are critical for recycling. When these dileucine motifs were mutated or when a dominant negative dynamin mutant was coexpressed, GLUT6 and GLUT8 were targeted to the plasma membrane [233]. Different to GLUT4/Glut4, plasma membrane targeting of these transporters could not be induced by insulin. A recent study suggests that GLUT6/Glut6 in the ME is involved in the regulation of glucohomeostasis [258]; however, the physiological and pathophysiological roles of GLUT6/Glut6 in brain remain elusive. The distribution of GLUT6/Glut6 in brain outside the hypothalamus has not been determined and it has not been elucidated under which condition GLUT6/Glut6 is targeted to the plasma membrane.

### GLUT8

When human GLUT8 was expressed in HEK293 or COS7 cells, the transporter was located within intracellular compartments; however, GLUT8 was targeted to the plasma membrane when a N-terminal dileucine motif was mutated [104, 178, 233]. After the expression of the dileucine mutant of GLUT8 in *Xenopus laevis* oocytes, uptake of 2DOG was obtained and a *K*_m_ value of 2.4 mM was determined [178]. Uptake of 2DOG into oocytes was partially inhibited by d-fructose and d-galactose. After reconstitution of wildtype GLUT8 in proteoliposomes, uptake of d-glucose was demonstrated [104]. In addition, evidence was provided that mouse Glut8 accepts the disaccharide trehalose as substrate [262].

GLUT8/Glut8 is ubiquitously expressed in humans and rodents [57, 104, 178]. GLUT8/Glut8 mRNA was abundantly detected in testis and less abundantly in skeletal muscle, spleen, heart, prostate, placenta, adipose tissue, adrenal gland, and brain. In human brain, GLUT8 mRNA was observed in cerebellum, brainstem, hippocampus, and hypothalamus [178]. In rat brain, the distribution of Glut8 was studied in detail employing in situ hybridization and immunohistochemistry [179, 341]. The experiments revealed that Glut8 was ubiquitously expressed in neurons. Most abundant Glut8-related immunoreactivity was observed in amygdala, primary olfactory cortex, dentate gyrus, dorsal hypothalamic area, supraoptic nucleus, pituitary stalk, and posterior pituitary [179]. In dentate gyrus and hippocampus immunoreactivity of Glut8 was observed in granular and pyramidal cells, respectively [341]. In both regions, Glut8 was also detected in non-principal neuronal cells. The Glut8-related immunoreactivity in neurons was observed in cell bodies whereas the plasma membrane was not stained [341]. Immunohistochemical colocation experiments indicated that Glut8 is expressed in excitatory and inhibitory neurons but not in astrocytes or microglial cells [341]. In neurons, Glut8 and Glut3 were coexpressed showing different subcellular locations. Glut8 was observed in cell bodies and proximal dendrites whereas Glut3 was located to neuronal plasma membranes, dendrites, and neurites. Immunohistochemistry in mice revealed a ubiquitous location of Glut8 in neurons similar to rats but suggested different expression levels in individual brain areas [363]. In addition to neurons, GLUT8/Glut8 was also localized to intracellular compartments of epithelial cells covering the choroid plexus and to ependymal cells in human and mice [288, 292].

In cerebral neurons of rodents, in COS7 cells transfected with human GLUT8, in murine neuroblastoma cells transfected with mouse Glut8, and in PC12 cells transfected with myc-tagged rat Glut8, GLUT8/Glut8 was located in intracellular compartments and it was observed that insulin did not promote targeting of GLUT8/Glut8 to the plasma membrane [233, 341, 365, 375, 441]. At variance, in murine blastocyst cells, Glut8 was targeted to the plasma membrane during the insulin-induced morphological changes of the blastocysts [57]. The subcellular distribution of Glut8 was investigated in detail using PC12 cells that were transfected with rat Glut8 [441]. Performing colocalization experiments with compartment specific proteins, Glut8 was identified in endoplasmic reticulum (ER) but not detected in early endosomes. In another study, the intracellular locations of mouse Glut8 and human GLUT4 co-expressed in CHO cells were compared [18]. No colocalization of Glut8 and GLUT4 was detected in the basal state. In contrast to GLUT4, no distribution of Glut8 to the plasma membrane was observed after treatment with insulin. Plasma membrane targeting of Glut8 could also not be induced by the Ca^2+^ ionophore A-23187 and the phosphatase inhibitor okadaic acid. Furthermore, it was observed that Glut8 does not share recycling endosomal compartments with the transferrin receptor and that Glut8 was localized to late endosomes and lysosomes. The effect of experimentally induced hyperglycemia on subcellular location of Glut8 in hypothalamic neurons was investigated in normal rats and in rats with streptozotocin (STZ)-induced diabetes [329]. Employing electronmicroscopic immunolocalization and membrane fractionation, it was observed that Glut8 was present in the cytosol and associated with low-density membranes. In normal but not in diabetic animals, cytosolic Glut8 distributed to the ER in response to hyperglycemia.

The physiological role and pathophysiological impact of GLUT8/Glut8 in brain are not well understood. When Glut8 was removed in mice, the proliferation of granular cells in the gyrus dentatus was increased [273]. The Glut8 knockout mice were hyperactive but showed no obvious effects in memory and explorative behavior [273, 364]. The data suggest that GLUT8/Glut8 is involved in energy supply for neurons in hippocampus [364]. It is however enigmatic how this is accomplished by a transporter located in the late endosome that may distribute to the ER. It has been discussed that GLUT8/Glut8 mediates the release of d-glucose that is generated during glycosylation of proteins from the ER; however, it is also possible that GLUT8/Glut8 transports d-glucose-6-phosphate into the ER during glucogenesis. Unfortunately, the substrate selectivity of GLUT8 has been poorly characterized so far. For example, the *K*_m_ for d-glucose uptake by wildtype human GLUT8 has not been determined and it has not been investigated whether GLUT8 accepts d-galactose, d-fructose, and phosphorylated monosaccharides as substrates.

### SGLT1

The Na^+^-d-glucose cotransporter SGLT1 (*SLC5A1*) is a secondary active transporter that translocates two sodium ions together with one molecule of d-glucose [446]. Human SGLT1 transports d-glucose and d-galactose with high affinity and efficacy. It transports 2DOG and 3OMG with low affinity but does not accept d-fructose as substrate (Table 1). Expressing human SGLT1 in oocytes and measuring monosaccharide uptake in the presence of physiological Na^+^ gradient and membrane potential, *K*_m_ values of 0.5 mM and 1 mM were determined for uptake of d-glucose and d-galactose, respectively [446]. In contrast to d-glucose and d-galactose, α-methyl-d-glucoside (AMG) is transported only by Na^+^-d-glucose cotransporters but not by GLUT transporters. Phlorizin is a high-affinity inhibitor of SGLT1 independently of species but does not inhibit GLUT transporters. Phlorizin also inhibits the Na^+^-d-glucose cotransporter SGLT2/Sglt2 of different species and blocks SGLT3/Sglt3b receptor functions in different species [446]. Porcine SGLT3 and the rodent subtype Sglt3b are Na^+^-d-glucose cotransporters whereas human SGLT3 and rodent Sglt3a are glucose sensors that do not transport monosaccharides [446]. For inhibition of human SGLT1 by phlorizin, *K*_i_ values around 200 nM have been determined [446].

SGLT1/Sglt1 is most abundantly expressed in small intestine and kidney [446]. In addition, SGLT1/Sglt1 is expressed in various organs, where it is partially located in rarely occurring structures. SGLT1/Sglt1 is expressed in heart, skeletal muscle, lung, liver, gall bladder, colon, rectum uterus, testes, pancreas, and brain [210]. SGLT1/Sglt1 mRNA in brain was observed in human, pig, rabbit, rat, and mouse [110, 118, 227, 290, 305, 330, 366].

By in situ hybridization in brains of rabbit and pig, SGLT1/Sglt1 was localized to cortical neurons, hippocampal pyramidal cells, and cerebellar Purkinje cells [110, 330]. In rat, Sglt1 mRNA was demonstrated in neurons of the VMH [118, 305]. In pig and rat, neuronal locations of SGLT1 expression were confirmed by immunohistochemistry [22, 330, 460]. SGLT1/Sglt1 may be also expressed in glial cells because Sglt1 mRNA was observed in primary cultures or rat astrocytes [422] and Sglt1-related immunoreactivity was reported in glial cells of the VMH [118]. The physiological importance of SGLT1/Sglt1 for glucose uptake into neurons was suggested by micro positron emission tomography (PET) and ex vivo autoradiography experiments was performed in rats [446, 459, 460]. In these experiments, an accumulation of α-methyl-4-deoxy-4-[18F]fluoro-d-glucopyranoside that is transported by Sglt1 and possibly also by Sglt2 but not by Glut1 and probably also not by other Glut transporters was observed in brain regions with high expression of Sglt1. For the PET experiments, the BBM had to be permeabilized.

Sglt1-related immunoreactivity was also observed in small vessels of rat brain [110]. After occlusion of the medial cerebral artery (MCAO) in rats, expression of Sglt1 in small brain vessels was also detected by in situ hybridization [110]. Evidence for the expression of (a) Na^+^-d-glucose cotransporter(s) in microvessels of brain was provided by transport measurements [224]. In this study, microvessels were isolated from bovine brain and luminal and abluminal membranes of the endothelial cells were isolated. Sodium-dependent, high-affinity uptake of d-glucose was observed in vesicles formed from abluminal membranes in contrast to vesicles of luminal membranes. Employing a different antibody against Sglt1 than Elfeber and coworkers [110] for immunohistochemistry in rat brain, Yu and coworkers did not detect Sglt1-related immunoreactivity in small blood vessels [460]. Although it cannot be excluded that Elfeber and coworkers observed nonspecific peptide blockable immunostaining of small blood vessels, it is more probable that Sglt1 did not show up in a slim, little prominent structural element under the experimental conditions employed by Yu and coworkers.

The expression of (a) Na^+^-d-glucose cotransporter(s) in the abluminal membrane of capillary endothelial cells suggests that SGLT1/Sglt1 and/or SGLT2/Sglt2 is(are) involved in the removal of d-glucose from brain interstitium where the concentration of d-glucose is 2–3 times lower than that in the blood [165]. In addition, SGLT1/Sglt1-mediated d-glucose uptake into neurons and an intracellular glucose sink due to glucose metabolism SGLT1/Sglt1 may contribute to the removal of d-glucose from brain interstitium. SGLT1/Sglt1-mediated removal of d-glucose from brain interstitium may be important to prevent glucotoxicity to neurons during reperfusion after brain ischemia. An exclusive expression of (a) Na^+^-d-glucose cotransporter(s) in the abluminal membrane of capillary endothelial cells provides an explanation why glucose analogs that are transported by SGLT1/Sglt1 but not by GLUT transporters such as ω-18F-fluoro-n-ethyl-β-d-glucosides and α-methyl-4-deoxy-4-[18F]fluoro-d-glucopyranoside do not pass the BBB and do not enter the brain [87, 459].

Taken together, the data show that SGLT1/Sglt1 is expressed in neurons throughout the brain showing high expression in regions that are involved in learning, regulation of feeding behavior, energy expenditure, and glucohomeostasis. Expression of SGLT1/Sglt1 in the BBB may be involved in adjustment of the glucose concentration in brain interstitium. The role of SGLT1/Sglt1 during diseases is enigmatic. In mice, cognitive impairment combined with damage of hippocampal neurons observed after chronic hypofusion was blunted when Sglt1 was removed [183], and a decreased cerebral expression of Sglt1 was protective during experimental TBI [366].

### SGLT2

The Na^+^-d-glucose cotransporter SGLT2/Sglt2 operates with a sodium/d-glucose stoichiometry of one [446]. Human SGLT2 transports d-glucose and AMG with *K*_m_ values around 5 mM but translocates d-galactose with very low efficacy (Table 1) [446]. SGLT2/Sglt2 is almost exclusively expressed in kidney; however, minor expression was also observed in brain [62, 113, 296, 360, 397, 446]. In human brain, SGLT2 mRNA was detected by RT-PCR where it appears to be most strongly expressed in cerebellum [62, 296, 397, 446]. In a proteomic analysis on microvessels isolated from rat brain cortex, expression Sglt2 was indicated [113]. Because the expression of SGLT2/Sglt2 in brain is very low and no data showing positive SGLT2/Sglt2-related signals in immunohistochemistry or in situ hybridization have been reported, the physiological relevance of SGLT2/Sglt2 in brain is questionable.

### SGLT3

Whereas one SGLT3 entity is expressed in human and pig, two subtypes called Sglt3a and Sglt3b have been cloned from rat and mouse [5, 25, 96, 243]. SGLT3 of pig and Sglt3b of mouse are Na^+^-d-glucose cotransporters which also accept AMG as substrate and are inhibited by phlorizin [5, 243, 446]. For d-glucose uptake by porcine SGLT3 and mouse Sglt3b, *K*_m_ values of 8 mM and 65 mM were determined [5, 446]. Human SGLT3 is a glucose sensor that induces membrane depolarization in response to low-affinity, phlorizin inhibitable binding of d-glucose and AMG by opening a channel-type Na^+^ and H^+^ permeability [96]. For d-glucose-induced membrane permeability of human SGLT3, *K*_0.5_ values between 20 and 60 mM were determined [96, 428]. At variance to human SGLT3 and mouse Sglt3a, rat Sglt3a exhibits a sodium-independent channel activity that is activated by d-glucose and AMG but cannot be blocked by phlorizin [25].

In human, SGLT3 mRNA was abundantly expressed in skeletal muscle but was also observed in various other tissues including adrenal gland, testis, uterus, small intestine, spinal cord, and brain [96, 296]. In rat hypothalamus and cultivated hypothalamic neurons, mRNAs of Sglt3a and Sglt3b were detected [305]. The expression of Sglt3a and Sglt3b in hypothalamic neurons suggests that SGLT3/Sglt3a play a role for activation of glucosensitive neurons by high d-glucose concentrations.

## Roles of glucose transporters in central nervous regulations of glucose homeostasis

### Overview

**Fig. 5:**
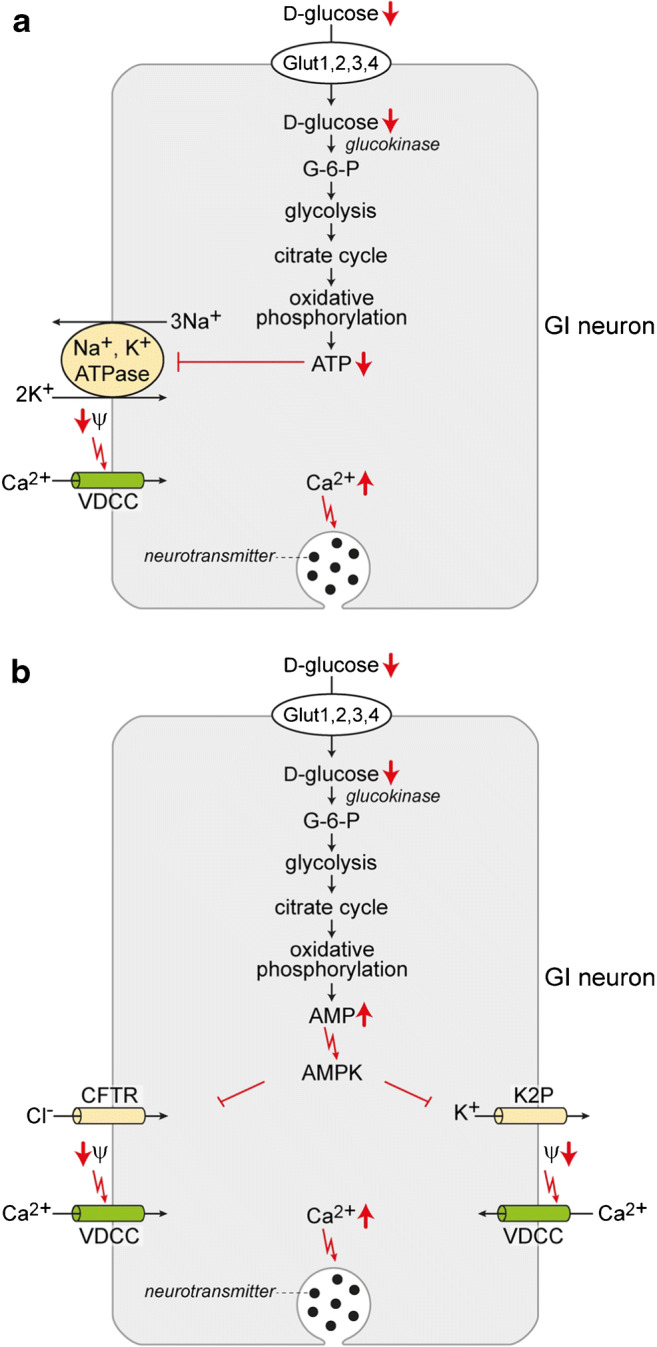
Involvement of glucose transporters in d-glucose sensing by neurons that are deactivated by d-glucose (GI neurons). Metabolism-dependent mechanisms detected in rodents are depicted in which a decrease of the extracellular d-glucose concentration leads to reduced d-glucose uptake by the glucose transporters Glut1, Glut2, Glut3, and/or Glut4. Decreased intracellular d-glucose promotes changes in metabolism resulting in a decrease and increase of intracellular ATP and AMP, respectively. **a** A mechanism based on the decrease of intracellular ATP is shown. Due to decreased intracellular ATP, the activity of the Na^+^-K^+^ATPase is reduced. This leads to a depolarization of the plasma membrane. The depolarization activates VDCC leading to an increase of intracellular Ca^2+^ that promotes neurotransmitter release. **b** Two mechanisms that are promoted by the increase of intracellular AMP activating AMP-dependent kinase AMPK are shown. Activation of AMPK may lead to a depolarization of the plasma membrane by blocking the chloride channel CFTR or the two-pore-domain potassium channel K2P. Opening of VDCCs leads to an increase of intracellular Ca^2+^ that triggers neurotransmitter release

Homeostasis of d-glucose in the blood is of fundamental importance for maintenance of physiological functions and health. Hypoglycemia that may occur during fasting and during treatment of diabetes with insulin must be avoided to maintain intact cerebral functions. Permanent low blood glucose levels may lead to damage of various organs including brain while permanent hyperglycemia causes microvascular complications such as nephropathy and macrovascular diseases including heart attack and stroke. Glucose homeostasis is regulated by peripheral mechanisms that are under central nervous control and by central steering of behavioral traits such as feeding behavior. The peripheral regulatory mechanisms include pancreatic secretion of insulin and glucagon. The central regulations are driven by glucose-sensitive neurons that are located in nuclei in the hypothalamus and brain stem. These neurons contain d-glucose-sensing mechanisms in which GLUT transporters, Na^+^-d-glucose transporters, the glucose sensors SGLT3/Sglt3a, or taste receptors may be involved (Figs. 4 and 5). d-Glucose-sensitive neurons have been identified in VMH, the dorsomedial hypothalamic (DMH) nucleus, the lateral hypothalamic area (LHA), the ARH, the nucleus of the solitary tract, and the dorsal vagal complex [10, 51, 84, 272, 306, 349]. d-Glucose-exitated (GE) and d-glucose-inhibited (GI) neurons have been distinguished [10, 307]. They have been shown to trigger regulations in response to hyper- and hypoglycemia by activating neuronal circuits that involve sympathetic and parasympathetic neurons [240, 387]. Under hyperglycemic conditions, GE neurons in VMH and the nucleus of the solitary tract that release GABA, and GE neurons in ARH that release anorexigenic peptides, are activated [42, 199, 316]. When the d-glucose concentration in the blood decreases, GI neurons in the LHA releasing orexin/hypocretin, and GI neurons in the VMH releasing glutamate and noradrenaline, were activated [52, 376, 403]. Sympathetic GI neurons in the VMH are blocked under hyperglycemic and hyperinsulinemic conditions [97].

### Sensing of blood glucose in brain

**Fig. 6:**
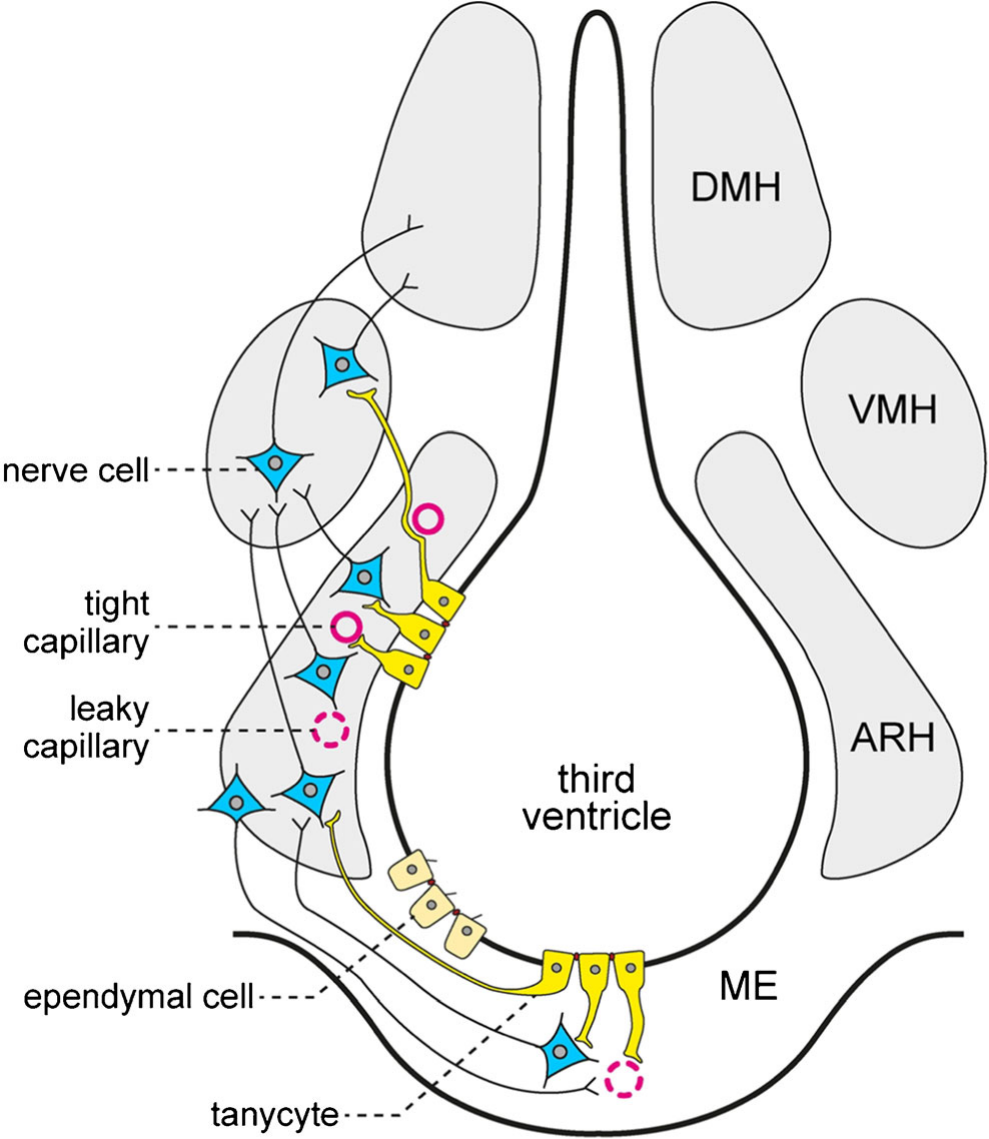
Locations of neurons, tanycytes, and ependymocytes in respect to brain ventricles, CVOs, and brain capillaries allowing glucose sensing in blood, CSF, and brain interstitium. The tuberal region of the hypothalamus with a CVO in the median eminence is depicted. Tanycytes sense the glucose concentration in the CSF within the brain ventricle and activate neurons. In addition, tanycytes and neurons sense the interstitial concentration of d-glucose close to leaky capillaries located in CVOs and the arcuate hypothalamic nucleus. Neurons also sense glucose concentrations in brain interstitium. Tanycytes are also supposed to be involved in the transfer of glucose from regions close to leaky capillaries and from capillaries of the BBB to the CSF. DMH dorsomedial nucleus, VMH ventromedial hypothalamic nucleus, ARH arcuate hypothalamic nucleus, ME median eminence

The interstitial d-glucose concentrations in most brain regions is only 10–30% of the d-glucose concentration in blood. d-Glucose in brain interstitium only changes slowly in response to blood glucose varying between 0.5 and 2.5 mM during the diurnal cycle [51, 354, 378]. Some hypothalamic neurons can sense the relatively low d-glucose concentration in brain interstitium and are supposed to be involved in slow and/or local regulations [51, 454]. At variance, rapid central nervous regulation of glucose homeostasis is based on sensing of d-glucose concentrations in the blood or in the CSF. d-Glucose sensing in the blood is achieved in two ways: on the one hand, by sensing blood glucose in tight capillaries by tanycytes, and on the other hand, by glucose sensing in CVOs with leaky capillaries via neurons and tanycytes (Fig. 6) [191, 333, 351]. Tanycytes that line brain ventricles send projections to neurons in hypothalamic nuclei. These projections may also contact tight brain capillaries of the BBM (Fig. 6). Other tanycytes have projections to leaky capillaries in CVOs. The d-glucose concentration in the CSF is similar to d-glucose in the blood. It changes rapidly in proportion to changes of blood glucose and may rise up to 15 mM during hyperglycemia [295, 389]. There is a controverse discussion whether d-glucose enters the CSF by passing the epithelial cells of the choroid plexus via GLUT/Glut transporters or via transcellular movement through tanycytes that connect cerebral ventricles with leaky and tight brain capillaries (Fig. 6) [240, 258]. In the epithelial cells of the choroid plexus, expression of Glut1, Glut4, Glut5, and GLUT8 was observed, and the location Glut1 was assigned to basolateral membranes (Table 3) [39, 102, 107, 155, 288, 406, 421]. In tanycytes, expression of Glut1, Glut2, Glut5, and Glut6 was detected [132, 212, 258].

In several areas close to hypothalamic nuclei and CVOs, brain ventricles are lined by tanycytes [128, 240, 333]. The β1 subgroup of the tanycytes is supposed to be specifically involved in the transmission of d-glucose-related signal to neurons in hypothalamic nuclei (Fig. 6) [287, 351]. d-Glucose sensing in tanycytes is performed by metabolism-dependent and metabolism-independent mechanisms. Metabolism-dependent sensing is supposed to involve GLUT/Glut transporter-mediated d-glucose uptake leading to an increase of d-glucose metabolism that results in elevated intracellular concentrations of ATP and l-lactate. Metabolism-independent d-glucose sensing in tanycytes may involve the sweet taste receptor T1R2/3 [240]. Both sensing mechanisms promote cellular release of ATP via connexin 43 hemichannels [128, 240, 309]. During metabolism-dependent d-glucose sensing, l-lactate is released from the tanycytes. It is hypothesized that extracellular ATP activates nucleotide receptors on tanycytes and neurons and promotes intracellular Ca^2+^ fluctuations that increase firing activity in neurons [128, 240]. Extracellular l-lactate may be taken up by neurons, enter citric acid cycle and oxidative phosphorylation, and increase intracellular ATP that may promote neuronal firing.

#### Mechanisms for glucose sensing in neurons

Several mechanisms are involved in d-glucose sensing in neurons (Figs. 4 and 5). An increase or decrease of extracellular d-glucose concentrations may induce depolarization in GE or GI neurons. Metabolism-dependent D-glucose sensing involving GLUT/Glut transporters and metabolism-independent glucose sensing involving SGLT/Sglt transporters, the glucose sensor SGLT3/Sglt3a, or the heteromeric sweet receptor T1R2/3 are distinguished.

The most abundantly discussed d-glucose-sensing mechanism that causes a cellular depolarization in response to increased extracellular d-glucose is analogous to the mechanism by which increased blood glucose stimulates insulin secretion in pancreatic β cells (Fig. 4a). This mechanism is dependent on metabolism. It comprises cellular d-glucose uptake mediated by a GLUT/Glut transporter, phosphorylation by pancreatic glucokinase (GK) that initiates glycolysis, followed by oxidative phosphorylation and blockage of an octameric ATP-sensitive K^+^ channel. The resulting depolarization of the plasma membrane triggers opening of voltage-dependent Ca^2+^ channels. The subsequent increase of intracellular Ca^2+^ leads to insulin secretion in pancreatic β cells and to neurotransmitter release in neurons [240]. As prerequisites for proper functioning of this sensing mechanism, several conditions must be met. The *K*_m_ for d-glucose uptake by the involved GLUT transporter must be higher than the sensed glucose concentration; e.g., the low-affinity GLUT2/Glut2 transporter is suitable for sensing blood glucose whereas the high-affinity GLUT1/Glut1 and GLUT3/Glut3 transporters are appropriate to sense glucose concentrations in brain interstitium. In addition, the synthesis of ATP must correlate with GLUT/Glut-mediated change of intracellular d-glucose and the ATP-inhibited open probability of the K^+^ channel must be decisive for membrane potential adjustment.

Cellular depolarization in response to increase of extracellular glucose can be also induced via mechanisms that are independent of metabolism. One mechanism involves d-glucose binding to the heterodimeric metabotropic sweet receptor T1R2/3 and activation of intracellular signal cascades that finally increase neuronal activity [240]. This mechanism may be associated with upregulation of SGLT1/Sglt1 expression [255]. The other metabolism-independent mechanism is based on functions of SGLT/Sglt proteins (Fig. 4b). It is due to the depolarizing effect of either sodium-coupled d-glucose uptake by a SGLT/Sglt transporter or to glucose-mediated activation of cation/proton permeability of a SGLT3/Sglt3a glucose sensor. Since human SGLT1 expressed in brain has a *K*_m_ value of 0.5 mM, this transporter senses low d-glucose concentrations. At variance, high d-glucose concentrations can be sensed by the human d-glucose sensor SGLT3 and the murine Na^+^-d-glucose cotransporter Sglt3b. Channel like activity of human SGLT3 was activated with *K*_0.5_ value between 20 and 60 mM [96] and mouse Sglt3b mediated d-glucose uptake with a *K*_m_ value of 65 mM [5]. An involvement of Sglt1 in neuronal d-glucose sensing in the VMH of rats was suggested by the observation that suppression of Sglt1 in VMH improved the counterregulatory increase of hepatic gluconeogenesis in response to recurrent hypoglycemia [118].

For depolarization in GI neurons in response to a decrease of extracellular d-glucose, three metabolism-dependent regulatory mechanisms involving GLUT/Glut transporters were distinguished. In rat, one mechanism was identified in the ARH that is involved in the regulation of feeding or blood glucose concentration in response to fasting [219] (Fig. 5a). Due to reduced d-glucose supply of ARH neurons, glycolysis, citrate cycle, and oxidative phosphorylation were slowed down causing a decrease of intracellular ATP and of Na^+^-K^+^-ATPase activity [219, 240]. The resulting decrease of intracellular K^+^ promoted a decrease of membrane potential that triggered Ca^2+^ influx. A second mechanism for activation of neurons by decreased extracellular d-glucose has been described for GI neurons in the VMH of mice [123] (Fig. 5b). Reduction of extracellular d-glucose leading to a decreased intracellular d-glucose concentration and an increased AMP/ATP ratio promoted the activation of AMPK. AMPK-induced closure of the chloride channel CFTR resulted in neuronal depolarization that triggered neuronal neurotransmitter release. The third mechanism was detected in mice for Glut2 expressing neurons in the nucleus of the solitary tract [222] (Fig. 5b). In this mechanism, the increase of the AMP/ATP ratio in response to decreased extracellular glucose-induced activation of AMPK that mediated closure of two-pore-domain potassium (K2P) channels in GABAergic neurons.

Finally, evidence for a metabolism-independent mechanism for d-glucose-mediated inhibition of orexin/hypocretin neurons in the LHA of mice has been provided [52]. These neurons are involved in regulation of awakefulness and metabolism. When extracellular d-glucose was increased from 1 to 2.5 mM, firing was blocked involving opening of a K2P channel that contains a TASK3 subunit. It was demonstrated that d-glucose acts from extracellular without changing intracellular concentrations of ATP and Ca^2+^.

#### Analysis of d-glucose sensing in defined neurons

In rodents, d-glucose sensing was demonstrated and characterized in some defined neurons. It was observed that neurons in the VMH that can be excited or blocked by leptin were also excited or inhibited by d-glucose [181]. In the hypothalamus, orexin neurons were identified that stopped firing when extracellular d-glucose was increased [444]. Part of these neurons adapted to increased ambient d-glucose concentrations within a second. It was also shown that d-glucose inhibited hypothalamic neurons that express agouti-related protein and neuropeptide Y [71, 286]. In addition, it was observed that d-glucose stimulated hypothalamic pro-opiomelanocortin expressing neurons [71, 180] and neurons expressing melanin concentrating hormone [214]. In individual neurons, different regulatory mechanisms for d-glucose sensing may be effective. Thus, different mechanisms were identified in GI neurons of the ARH [219], the VMH [123, 277], the NTS [222], and the LHA [52]. In GE neurons of rat hypothalamus, two glucose sensing mechanisms were distinguished [305]. A relatively small fraction of the GE neurons was activated by a metabolism-dependent mechanism involving a Glut transporter and an ATP-dependent K^+^ channel (Fig. 4a) whereas the predominant fraction of the GE neurons was activated by the metabolism-independent mechanism involving a phlorizin inhibitable Sglt transporter or a phlorizin inhibitable Sglt-type glucose sensor (Fig. 4b) [305].

### Regulation of whole-body glucose homeostasis in brain

#### Regulation of insulin and glucagon secretion

Pancreatic secretion of insulin and glucagon are not only regulated in pancreas in response to d-glucose in the peripheral blood but also in the hypothalamus in response to d-glucose in CSF or brain capillaries. The central nervous regulation is supposed to involve GLUT2/Glut2 and GLUT4/Glut4 in CVOs and/or hypothalamic nuclei. When d-glucose in rodent brain was increased by injection of a small amount of d-glucose into the carotic artery without changing the d-glucose concentration in the peripheral blood, pancreatic insulin secretion was increased [153, 232]. This effect was associated with d-glucose-induced activation of hypothalamic nuclei and blunted when the metabolism of glial cells was blocked [153]. Central nervous stimulation of pancreatic glucagon secretion in response to hypoglycemia was shown to depend on the expression of Glut2 in glial cells [259]. Central nervous regulation of blood glucose was also demonstrated in mice in which the cerebral expression of Glut4 was abolished (GB4KO mice) [346]. In these mice, d-glucose uptake into the ARH and VMH was reduced. The GB4KO mice showed an increased rise in blood glucose during an intraperitoneal glucose tolerance test (IGTT) although the accompanying rise in blood insulin, peripheral glucose disposal, and the insulin tolerance test (ITT) were similar to wildtype mice [346]. The data suggest that the central nervous suppression of endogenous glucose production (EGP) in response to increased blood glucose was decreased after removal of Glut4 in brain.

#### Regulation of feeding behavior

Feeding behavior and appetite are regulated by hypothalamic networks in neurons of the VMH, LHA, and ARH that are sensitive to circulating and local signal molecules including leptin, ghrelin, neuropeptide Y, orexin, insulin, and d-glucose [51, 133, 181, 388, 416, 449]. Regulation of feeding behavior is frequently associated with central nervous regulation of insulin and glucagon secretion. When blood glucose decreased during fasting, EGP and food intake was increased in rodents. These regulations involved glucose sensors in neurons and tanycytes that register d-glucose concentration in blood and CSF (Fig. 6) [68, 69]. In rats, food intake was induced when the antiglycolytic agent 5-thioglucose or the GK inhibitor alloxan were injected into the fourth brain ventricle [347, 348]. Both compounds are inhibitors of metabolism-dependent d-glucose sensing. Similarly, food intake was induced after injection of the d-glucose-depriving monosaccharide 2DOG [279]. Indicating an involvement of metabolism-independent d-glucose sensing in regulation of feeding behavior, food intake was induced in rats after i.c.v. injection of the SGLT/Sglt inhibitor phlorizin [139, 404]. Also sweet taste receptors in tanycytes may be involved since it was observed that tanycytes sense ligands of the Tas1r2/Tas1r3 sweet receptor and that the proportion of d-glucose-sensitive tanycytes was decreased in Tas1r2 knockout mice [29].

Glut2 and Glut4 are involved in the regulation of feeding behavior in response to blood glucose in rodents. In rodents, expression of Glut2 was observed in CVOs, tanycytes, and hypothalamic nuclei, whereas expression Glut4 was observed in hypothalamic nuclei. When Glut2 expression was decreased by injection of Glut2 antisense RNA into the third brain ventricle of rats, the stimulation of food intake in response to intraventicular injection of 2DOG was blunted [430]. Genetic suppression of Glut2 in tanycytes provided evidence that Glut2-dependent glucose sensing in tanycytes is critical for d-glucose-dependent regulation of feeding behavior [24]. After ablation of Glut4 expressing neurons in hypothalamus of mice, food intake was largely decreased [345].

## Regulation of glucose transporters in response to neuronal activity

### Introduction

Brain is nearly exclusively fueled by d-glucose. To minimize glucotoxic effects on neurons, the concentration of d-glucose in brain interstitium is adjusted to about 20% of blood glucose. Glucose supply of brain under resting conditions is adjusted to a level that is only just sufficient [26, 381]. Local energy demand is largely increased during neuronal activation, for instance during sensory stimulation, exercise, and mental activity, because much energy is consumed during excitation of neurons and synaptic neurotransmission [162]. Architecture, functional properties, and regulatory mechanisms in the BBB, astrocytes, and neurons allow an efficient provision of energy during neuronal activation (Figs. 1 and 3) [195]. Most d-glucose leaving brain capillaries is taken up by astrocytes that path d-glucose or its glycolytic metabolite l-lactate, to neurons. Some d-glucose may leave brain capillaries via diffusion through gap junctions that connect end-feet of astrocytes and may enter neurons directly. The regulation of d-glucose transport in response to neuronal activation via GLUT1/Glut1 in capillary endothelial cells and astrocytes and via GLUT3/Glut3 and/or Glut4 in neurons has been investigated in detail.

### Regulation of d-glucose transport across the BBB

The regulation of d-glucose transport across the BBB in response to neuronal activity involves regulation of blood supply, adjustment of driving forces for transport, and regulation of the expression of glucose transporters. In response to neuronal activation, blood flow in rat brain capillaries was shown to be increased due to dilatation of arterioles [142]. In addition, the d-glucose concentration gradient between blood and brain interstitium that represents the driving force for d-glucose transport across capillary endothelial cells mainly mediated by GLUT1/Glut1 is maintained. This driving force is generated by d-glucose uptake into astrocytes and neurons via GLUT/Glut transporters. The d-glucose uptake into astrocytes and neurons is driven by intracellular d-glucose phosphorylation that keeps the concentration of free intracellular d-glucose low. During neuronal activation, d-glucose phosphorylation is accelerated. It has been observed that Glut1 in plasma membranes of capillary endothelial cells was upregulated during neuronal activation and proposed that this upregulation of Glut1 is mediated by paracrine activation via astrocytes [9, 37, 342]. Although pathological conditions such as epileptic seizures or hypoglycemia lead to more severe energy depletion in neurons compared to physiological neuronal activation, similar regulatory processes and mechanisms may be involved. Thus, d-glucose transport across the BBB was upregulated after a seizure and in glucose depleted cultured endothelial cells [79, 82]. Three minutes after induction of a seizure by pentylene tetrazole in rats, the rate of 2DOG removal from brain vessels was increased 30–40% and this effect was due to an increased *V*_max_ [79]. After ATP depletion of endothelial cells derived from small brain vessels of rats, Glut1 was incorporated into the plasma membrane and *V*_max_ of glucose uptake was increased [82]. This effect was mediated by activation of AMPK which phosphorylates thioredoxin interacting protein TXNIP that binds to Glut1 [447].

### Impact of interplay between astrocytes and neurons

During neuronal activation, membrane abundance of Glut1 and glycolysis in astrocytes were upregulated within seconds leading to an interstitial decrease of d-glucose and increase of l-lactate [55, 173, 176, 334, 378]. In addition, membrane abundance of GLUT3/Glut3 and/or GLUT4/Glut4 may be increased in neurons [16, 382]. In activated neurons, stimulation of Glut1 mediated d-glucose uptake into astrocytes was induced within seconds whereas d-glucose uptake into neurons was not affected [67, 239, 331]. This rapid upregulation of Glut1 in astrocytes was mainly mediated by uptake of released glutamate via the Na^+^-glutamate cotransporter that leads to an increase of intracellular Na^+^ which triggers increase of intracellular Ca^2+^ [331]. Glycolysis in astrocytes in response to neuronal activation is rapidly stimulated by two mechanisms [435]. In one mechanism, increased intracellular Na^+^ due to Na^+^ coupled glutamate uptake stimulates the Na^+^-K^+^-ATPase that leads to an increased ATP hydrolysis resulting in a decreased ATP/(ADP + AMP) ratio. The increase of AMP promotes allosteric activation of glycolytic enzymes [60]. In the other mechanism, the increased concentration of interstitial K^+^ in response to neuronal activation induces a depolarization of astrocytes. The depolarization activates HCO_3_^−^ uptake by the electrogenic Na^+^/HCO_3_^−^ cotransporter NBCe1 leading to an intracellular alkalization that stimulates glycolytic enzymes [35, 358].

Neurons isolated from rat hippocampus were cultivated in the presence of insulin and the effect of activation on membrane trafficking of Glut3 and Glut4 was investigated [16]. In response to neuronal activation, Glut4 was rapidly inserted into the plasma membrane whereas plasma membrane abundance of Glut3 was not altered. The plasma membrane insertion of Glut4 containing vesicles was shown to be triggered by activation of AMPK.

### Effects of sustained neuronal activations

#### Exercise

During exercise, the l-lactate concentration in brain increases due to activation of the astrocyte-neuron lactate shuttle that provides additional energy to activated brain regions. Several hours after one bout of exercise or after prolonged exercise in rodents, plasma membrane expression of Glut1 and/or Glut4 was(were) changed in distinct brain regions [6, 21, 393]. Thirty minutes after 2-h exercise of mice on a treadmill, l-lactate concentrations in hippocampus and brain cortex were increased [393]. Five hours after exercise, expression of lactate transporters in brain vessels, astrocytes, and neurons was upregulated whereas Glut1 in brain cortex was upregulated only after 18 h. In another study performed in mice, the effect of exercise on glucose transporters in cerebellum was investigated [21]. After 2-h exercise on a treadmill, uptake of FDOG in cerebellum and the abundance Glut4 protein in cerebellar plasma membranes were increased. In cultivated cerebellar neurons, it was demonstrated that insulin stimulated plasma membrane insertion of Glut4 [21]. In addition, Glut4 protein was colocalized with insulin-responsive aminopeptidase and with the putative sorting receptor sortilin.

#### Learning and memory

Neuronal circuits in hippocampus play pivotal roles in learning and memory formation representing processes that are associated with high energy consumption in neurons. The increased energy demand is met by provision of d-glucose and l-lactate in combination with increased aerobic glycolysis. The increased provision of d-glucose and l-lactate to neurons is accomplished by upregulation of glucose and lactate transporters and by increased glycolysis in astrocytes [293, 322, 323]. The upregulations are triggered by a decreased extracellular d-glucose concentration and by an increased cerebral secretion of insulin.

During training of rodents for different memory tasks, a decrease of d-glucose and an increase of l-lactate in hippocampus were measured [269, 270, 293, 392]. The cognitive effect of training was augmented when the decrease of cerebral d-glucose was prevented by provision of d-glucose [269, 270]. In addition to upregulation of lactate transporters in astrocytes and neurons during learning, upregulation of Glut1, Glut3, and Glut4 was observed [65, 322, 323, 392]. Furthermore, it turned out that the short-term memory was improved by phlorizin, an inhibitor of Sglt1, Sglt2, Sglt3a, and Sglt3b [38, 156].

Evidence was presented that brain-derived insulin that interacts with the insulin receptor in brain is involved in memory-related hypothalamic neuronal circuits [23, 268, 465]. Thus, application of insulin to hippocampus improved the performance of a spatial memory task in rats whereas the performance was impaired when endogenous insulin in the hippocampus was inactivated [271]. Training rats for an operative memory task increased hypothalamic expression of the insulin receptor [465]. Moreover, in rats with insulin resistance that had been induced by a high-fat diet (HFD), memory performance was impaired and the effect of hippocampal application of insulin was blunted [271]. Since it was observed that the administration of d-glucose improved memory performance similar to insulin [140, 267] and that insulin-stimulated plasma membrane insertion of Glut4 in hippocampal neurons [339], it was reasoned that the effect of insulin on memory performance may be mediated via upregulation of Glut4. Studying the role of Glut4 in learning that involves hippocampal neuronal circuits, spontaneous alteration (SA) operational memory tasks were employed [73, 322, 323]. In these experiments, it was ensured that insulin signaling in hippocampus was required for successful accomplishment of the tasks, that insulin administration to hippocampus improved the outcome, and that upstream components of insulin regulation were involved. It turned out that during short-term learning, glucose utilization in the dorsal hippocampus was increased and Glut4 abundance in hippocampal plasma membranes was upregulated whereas plasma membrane abundance of Glut1 and Glut3 was not changed [150, 323]. To determine the impact of Glut4-mediated glucose transport on learning, Glut4 in hippocampus was inhibited by the HIV drug indinavir that does not inhibit Glut1 and Glut3. Selective blockage of Glut4-mediated transport impaired the improved outcome in the SA operational task observed after hippocampal application of insulin [323]. The outcome in the SA operational task without application of insulin was impaired when the upstream pathway of insulin-dependent Glut4 trafficking was blocked [271, 323]. The data demonstrate the requirement of insulin-dependent upregulation of Glut4 during learning. In contrast to neuronal glucose uptake during learning, neuronal glucose uptake during unforced brain activity is supposed to be mainly covered by Glut3. Glut4 probably contributes, because prolonged inhibition of Glut4 in hippocampus led to upregulation of Glut3 that resulted in an improvement of the working memory [322]. The observation that the expression of Glut1 in hippocampus of mice was increased about 4 h after a conditioning task suggests that Glut1 is also is involved in learning [65]. The upregulation of Glut1 is supposed to be mediated by a cooperative effect of insulin and insulin growth factor 1 (IGF-1) on the expression of Glut1 in astrocytes involving a mitogen-activated protein kinase/protein kinase D pathway [122].

## Cerebral glucose transporters during diabetes

### Introduction

It has been reported that cerebral d-glucose uptake and expression of glucose transporters in brain change in response to consumption of HFDs and during type 1 and type 2 diabetes mellitus. HFDs promote obesity and type 2 diabetes mellitus (T2DM) that have been identified as risk factors for emergence of PD. In this chapter, changes in cerebral d-glucose transport and expression of glucose transporters in brain during type 1 diabetes mellitus (T1DM) and T2DM are described. Considering the impact of cerebral glucose transporters for operative learning and memory formation, also diabetes associated changes in cognitive functions are discussed.

### Type 1 diabetes mellitus

T1DM in which insulin secretion by pancreatic β cells is destroyed has been identified as risk factor for development of cognitive impairment in humans [170, 275, 355, 359] and in rodents with STZ-induced T1DM [33, 34, 320]. In humans with T1DM, similar cerebral d-glucose concentrations and d-glucose uptake rates into brain were observed under normo-, hypo-, and hyperglycemic conditions [119, 154, 415] suggesting that d-glucose-dependent regulation of glucose transporters is intact in T1DM. In rats, detailed investigations concerning effects of STZ-induced diabetes on cerebral d-glucose uptake and expression of glucose transporters in brain were performed. Some of the reported data are diverging. Measuring tracer uptake of radioactively labeled d-glucose, 2DOG or 3OMG into total brain or frontal cortex, a decreased or unchanged uptake was observed in rats with STZ-induced diabetes [196, 280, 336]. In small vessels isolated from total brain, upregulation of Glut1 mRNA was observed in the diabetic animals [66, 241] whereas the abundance of Glut1 protein was either downregulated [241, 314, 332] or not changed significantly [19, 280, 395]. In hippocampus of rats with STZ-induced diabetes, Glut1 mRNA was upregulated whereas Glut1 protein abundance was not changed [341]. At variance, Glut3 in hippocampus was upregulated on mRNA and protein level [340]. The upregulation of Glut3 is supposed to be specific for hippocampus since no upregulation was detected in total brain samples. In cerebellum of rats with STZ-induced diabetes, a higher abundance of Glu4 was observed compared to non-diabetic animals [420]. Finally, it has been described that in mice with STZ-induced diabetes, insulin-dependent translocation of Glut4 to plasma membranes of hippocampal neurons was affected [320]. In the diabetic mice, locomotion and cognitive functions were impaired. Taken together, the data suggest that changes of Glut4 and Glut3 mediated glucose uptake into hippocampal and cerebellar neurons are associated with cognitive and operational impairments during T1DM.

### Type 2 diabetes mellitus

#### High-fat diets and obesity as precursors of type 2 diabetes

Prolonged consumption of hypercaloric HFDs, in particular of HFDs that contain large amounts of sucrose, so called Western diets, lead to obesity, induce metabolic changes including insulin intolerance, and promote T2DM [117, 260]. Peripheral and central insulin intolerance are key symptoms of T2DM beside increased concentration of d-glucose in blood and impaired pancreatic insulin secretion. It has been observed in humans and rodents that prolonged nutrition with hypercaloric HFDs leads to impaired hippocampus-related memory functions that are associated with insulin resistance [115, 127, 146–148, 200, 284, 414]. The impairments are influenced by the abundance of saturated fatty acids and sucrose in the diet [146, 147]. The effects of HFDs on memory functions have been studied in rat models for T2DM using different operative memory tests [73]. For example, some impairment of the working memory measured in a radial maze test was observed when rats were kept for 9 days on a hypercaloric HFD, although body weight and morning blood glucose were not increased [289]. When rats were kept for 3 months on HFD, they became obese, exhibited peripheral and central insulin resistance and dysfunction of hippocampal mitochondria. These rats showed an impairment of the operative spatial memory measured by the Morris water maze task [73, 327, 328]. The brain dysfunction was improved by antidiabetic drugs like metformin or dipeptiyl-peptidase 4 inhibitors [326–328]. Arnold and coworkers observed impaired insulin sensing in brain cortex and hippocampus of mice after feeding for 17 days with a hypercaloric HFD [15]. The mice had a 20% higher body weight and an increased morning blood d-glucose concentration compared to mice on control diet. Operative spatial memory in a T-maze task [73] was impaired. It is probable that Glut4 in hippocampus that participates in operational learning [323] is involved.

In mice, a transient impairment of an operational memory task correlated with downregulation of Glut1 has been described [190]. Administration of HFD for 3 days led to downregulation of Glut1 mRNA and protein in small vessels in different brain areas including cerebral cortex and hippocampus. The downregulation was reversed when the HFD was administered for 8 days or longer time periods. Downregulation of Glut1 observed after 3 days was associated with a decrease of d-glucose uptake in hippocampus and brain cortex. Evidence was presented that the reversal of Glut1 downregulation was induced by an increased expression of vascular endothelial growth factor in macrophages.

#### Type 2 diabetes mellitus in human

Correlations between deficits in memory functions and T2DM have been observed using various cognitive tests. Employing a psychological test battery, it turned out that speed of reaction time was decreased and the outcome in a memory-concentration task was impaired in patients with T2DM [275]. In a prospective study following T2DM patients over a period of about 30 years, increased risk for poor performance of tests on verbal memory was associated with duration and severity of T2DM [111]. In another large prospective multicenter study on women older than 64 years, the effect of T2DM on cognitive functions was tested three times every 3 years [149]. It turned out that T2DM was associated with impaired cognitive functions and accelerated cognitive decline.

#### Rodent models of type 2 diabetes

Individual aspects concerning effects of T2DM on cerebral glucose transporters were investigated in rodent models. The employed models were diet induced obesity (DIO) [271], *ob/ob* mice [213], *db/db* mice [419, 420], and Zucker diabetic fatty (ZDF) rats. When rats were fed for 20 days with HFD, part of the animals became obese and developed diabetes with increased blood d-glucose and blood insulin concentrations (DIO rats). In the DIO rats, memory performance measured in SA memory tasks was impaired compared to diet resistant rats or to rats on standard chow [271]. *Ob/ob* mice containing defect mutations in the leptin coding gene become obese and exhibit high blood levels of d-glucose and insulin. In the *ob/ob* mice, increased expression of Glut4 was observed in neurons of the ARH which is supposed to be involved in central regulation of blood glucose [213]. Adult *db/db* mice expressing a defective leptin receptor are obese and insulin resistant. They exhibit increased levels of d-glucose and insulin in the blood. In adult *db/db* mice, a decreased brain weight and decreased cerebral d-glucose utilization was observed [419]. In the *db/db* mice, protein abundance of Glut1 in the BBB and of Glut3 in total brain were similar to nondiabetic littermates [419]. In cerebellum of *db/db* mice, the expression of Glut4 protein was increased [420]. ZDF rats which exhibit a highly increased blood d-glucose concentration and an increased concentration of d-glucose in hippocampus showed a decreased abundance of Glut4 protein in hippocampal plasma membranes whereas tissue abundance of Glut4 protein in hippocampus was not altered [417, 445]. Performing interval learning tasks, memory functions with longer time intervals were impaired in ZDF rats [445]. Taken together, the data suggest that the decrease of mental functions observed in patients with badly controlled T2DM may be associated with changes in regulation of GLUT4 in hippocampus.

## Cerebral glucose transporters during Alzheimers’s disease

### Pathogenesis of AD

#### Overview

AD is the most abundant cause of progressive intellectual failure in aged humans [36, 369, 456]. Two types of AD are distinguished: early-onset AD that is observed in 5–10% of patients, often starts early in live and is caused by genetic abnormalities, and late-onset AD that is observed in 90–95% of patients. Late-onset AD mainly emerges in aged individuals and is supposed to be caused by complex interactions of genetic and environmental factors that provoke neuronal hypometabolism. Advanced stages of AD are characterized by extensive synaptic loss that is associated with decreased d-glucose uptake and d-glucose metabolism in specific brain areas [92, 368]. Early described hallmarks of neuronal damage during AD were neuritic extracellular amyloid plaques—called senile plaques (SPs)—and cytosolic neurofibrillar tangles (NFTs). The SPs are formed by amyloid beta-peptides (AβPs) that are fragments of beta-amyloid precursor protein (APP). NFTs are aggregates of abnormally hyperphosphorylated cytosolic tau protein. The occurrence of SPs and NFTs in early-onset and late-onset AD, and the observation that missense mutations in APP cause autosomal dominant forms of early-onset AD, gave rise to the so-called classical hypothesis on AD pathogenesis. This hypothesis states that AD is caused by formation of AβP oligomers that form SPs and induce neuronal injury promoting formation of NFTs. Whereas this pathogenetic mechanism may be valid for some forms of early-onset AD, late-onset AD is considered as a metabolic disease with hypometabolism in specific brain regions as described by the neuroenergetic hypothesis on AD pathogenesis. The hypometabolism can be induced by continuing or recurrent effects of genetic and/or environmental factors. In this chapter, both hypotheses for the pathogenesis of AD are outlined. In addition, associated structural and functional changes in brain associated with late-onset AD are described, and the potential role of glucose transporters in pathogenesis of AD is discussed.

#### Classical hypothesis on AD pathogenesis

In the classical hypothesis on AD pathogenesis, the formation of extracellular AβP oligomers is considered as initial event [14, 159, 160, 285, 344]. AβPs are derived from APP variants that are degraded by β- and γ-secretase and contain a part of the hydrophobic transmembrane domain of APP. Inherited forms of early-onset AD may be due to mutations in APP that block cleavage sites for secretases, to genetic variations in presenilin 1 and 2 that interact which γ-secretase and enhance the formation of AβPs, and to genetic variations in apolipoprotein E that is supposed to be involved in the clearance of AβP. AβP forms oligomers and SPs and promotes synaptic and neuritic injury. The neuritic injury is combined with changes of intracellular ionic homeostasis and kinase/phosphatase activities and leads to neuronal dysfunction and cell death. The changed kinase/phosphatase activities lead to hyperphosphorylation of microtubule-associated protein tau and formation of intraneuronal tangles. Whereas it is established that extracellular AβP oligomers can cause AD by exhibiting neuritic injury, the detailed mechanism promoting the injury is not understood. The observations that the occurrence of extracellular SPs and NFTs was not always correlated during AD and that the number of SPs in patients could not be unambiguously correlated with the degree of cognitive impairment, are in contradiction to a general validity of the classical hypothesis on AD [14, 285, 344].

#### Neuroenergetic hypothesis on AD pathogenesis

The ambiguity in the causal chain proposed by the classical hypothesis on AD pathogenesis and the inability of this hypothesis to comprise the multiple genetic and environmental factors that promote late-onset AD [14, 285] showed the demand of an alternative more general hypothesis. Thus, the neuroenergetic hypothesis was raised in which decreased metabolizable energy availability for neurons is the key factor of AD pathogenesis [36, 85]. A central observation leading to this hypothesis was that permanent or occasional insufficiency of energy supply is neurotoxic and leads to a destruction of synapses and neurons. This destruction may include intracellular signaling, inflammatory reactions, and microglial cell activities. It was observed that AD is always associated with a reduction of d-glucose uptake into specific brain regions that may be caused by decreased blood flow and/or decreased expression of glucose transporters in the BBB. The destruction of neurons in response to reduced energy supply may be influenced by various genetic and environmental factors, for example by apolipoprotein E4 and HFD [4, 169]. Likewise, the neuroenergetic hypothesis is consistent with the observations that the incidence for AD is increased with age and in patients with T1DM or T2DM [274, 311]. With increasing age and during diabetes, blood flow through small blood vessel, cerebral d-glucose uptake into brain, and cerebral glucose metabolism are impaired. During insulin-treated T1DM, hypoglycemia may promote neuronal destruction whereas insulin resistance during T2DM may decrease the insulin upregulation of GLUT4/Glut4 in hippocampal neuronal membranes [320].

### Cerebral uptake and metabolism of d-glucose during AD

#### Changes in utilization and metabolism of d-glucose

PET employing [^18^F]2-fluoro-2-deoxy-d-glucose ([^18^F]DOG) allows the identification of brain regions with decreased uptake and/or phosphorylation of d-glucose [89, 105, 129, 167, 188, 383]. Like [^14^C]DOG that had been introduced in 1977 for autoradiographic studies in animals [386], [^18^F]DOG serves as tracer for netto uptake of d-glucose from blood into brain followed by phosphorylation via hexokinase. [^18^F]DOG phosphorylated in position six ([^18^F]DOG-6-P) is not metabolized further and trapped in cells. In addition to accumulation of [^18^F]DOG-6-P in brain tissue after a defined time period, also the time course of radioactivity accumulation can be determined. Employing simple models, time constants for uptake of [^18^F]DOG (k_1_), efflux of [^18^F]DOG (k_2_), phosphorylation of [^18^F]DOG (k_3_), and dephosphorylation of [^18^F]DOG-6-P (k_4_) were estimated [188, 325]. During AD, [^18^F]DOG-6-P accumulation was decreased in various brain regions. Distinct signal reductions were observed in the frontal, temporal, parietal, occipital, and entorhinal cortex, and in hippocampus [89, 167, 188, 325]. These regions overlap with brain regions in which the largest histological changes in response to AD were observed [46]. The degree of PET [^18^F]DOG signal reduction during AD was correlated with the severity and rate of progression of cognitive defects [89, 189]. Interestingly, reductions of [^18^F]DOG signals in non-diseased individuals were correlated with genetic risk for AD [343]. Model analysis of time courses of PET with [^18^F]DOG revealed that the time constants for [^18^F]DOG uptake (*k*_1_) and [^18^F]DOG phosphorylation (*k*_3_) were decreased [188, 325]. The decrease of k_1_ suggests a decelerated passage of d-glucose from blood capillaries into brain tissue. This can be due to slowed blood flow through small brain vessels, decreased d-glucose transport across the BBB, and/or decreased d-glucose transport into cerebral cells. The decreased *k*_3_ value indicates a decelerated metabolism of d-glucose. PET with [^18^F]DOG is a valuable tool for diagnosis of AD and evaluation of neuronal damage during AD.

#### Changes in blood flow

During AD and other dementing illnesses, expansion and architecture of small blood vessels is altered and cerebral blood flow is decreased [47, 125, 249, 357, 440]. Local functional and structural changes in microvessels are supposed to represent an early event during emergence of AD [335, 384]. Accordingly, the hypothesis was raised that hypoperfusion can be an early event in the causal chain of AD pathogenesis [242, 254, 361, 468, 470]. Accordingly, an impairment of regional blood flow was detected in patients during early stages of AD where no distinct tissue defects were detectable [335]. In addition, in cognitively intact individuals with genetic risk factors for AD, task activation of blood flow was impaired in brain areas in which neuropathological changes during AD have been described [384]. Furthermore, the impact of cerebral hypoperfusion on the pathogenesis of AD was suggested by experiments with rodents. It was observed that chronic cerebral hypoperfusion led to cognitive impairment and neurodegeneration in hippocampus that was associated with accumulation of AβP oligomers [432]. In a mouse model of AD, transient cerebral hypoperfusion induced an upregulation of AβP in brain [211]. All in all, impairment of blood supply represents one way how energy supply to neurons can be reduced. It may represent a starting point of AD pathogenesis according to the neuroenergetic hypothesis. In general terms, brain capillaries have high impact on emergence of cerebral malfunctions including AD. They are part of the neurovascular unit that does not only play a central role in regulation of local blood flow in response to neuronal activity but also in regulation of capillary growth and AβP transfer from the blood into brain tissue [91, 177, 469].

#### Changes in expression of glucose transporters

The expression of GLUT1, GLUT2, GLUT3, and GLUT4 in brain tissue and small brain vessels has been compared between patients with AD and healthy individuals [197, 236, 281, 379]. During AD, downregulation of GLUT1 protein was detected in cerebral cortex and hippocampus [236, 281, 379] and confined to GLUT1 in endothelial cells of brain capillaries [172, 197, 425]. In a mouse model for early onset of AD in which atrophy of hippocampus and increased Aβ abundance in hippocampus were observed whereas capillary density in hippocampus was not changed, Glut1 protein in small hippocampal vessels was decreased [171]. In patients with AD, also the cerebral abundance of GLUT3 protein was decreased [161, 236, 379]. Downregulation of GLUT3 was observed in cerebral cortex and hippocampus. It was due to downregulation of GLUT3 in neurons [379]. In brain tissue from AD patients, the expression of GLUT2 protein was upregulated whereas a similar expression of GLUT4 protein was observed as in non-diseased individuals [236].

### Potential roles of glucose transporters during AD pathogenesis

**Fig. 7:**
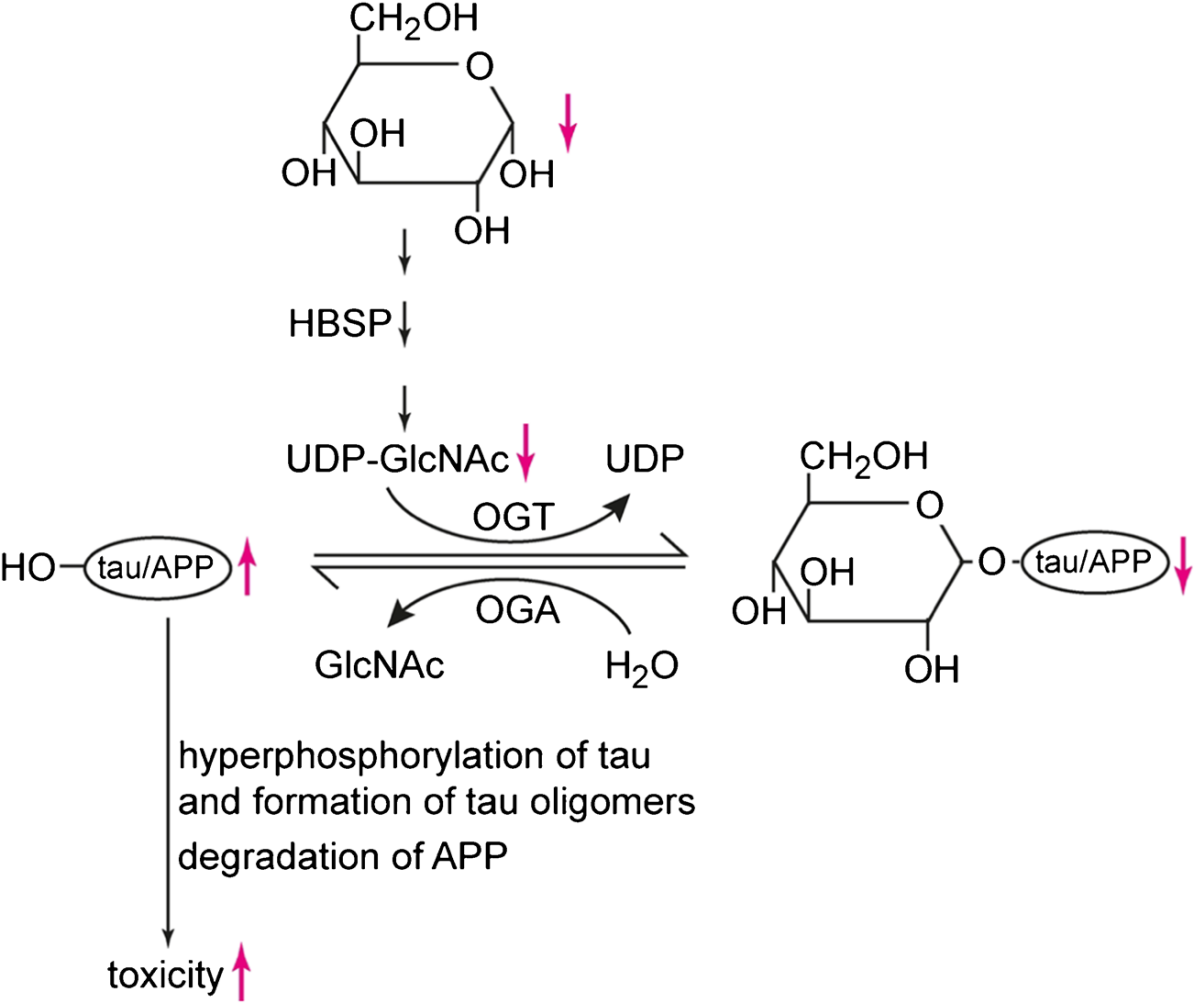
Hypothesis how decreased expression of glucose transporters in brain leading to a decreased intracellular d-glucose concentration in neurons may promote the emergence of AD. A reduced concentration of d-glucose in neurons decelerates the biosynthetic pathway of hexosamine (HBSP) leading to a decreased *O*-glycosylation of proteins tau and APP with *N*-acetylglucosamine. The glycosylation of these proteins is neuroprotective because it decreases hyperphosphorylation of tau that promotes the formation of tau oligomers and decreases Aβ formation by degradation of APP. The effects of downregulation of cerebral glucose transporters are indicated by red arrows. GlcNAc *N*-acetylglucosamine, UDP-GlcNAc uridine 5′-diphosphate-*N*-acetylglucosamine, OGT O-GlcNAc transferase, OGA O-GlcNAcase, APP amyloid precursor protein

The downregulation of GLUT1/Glut1 and GLUT3/Glut3 protein during AD could represent an early concomitant phenomenon that aggravates AD progression [466] or an early member within the causal chain of the pathogenetic mechanism of AD [151]. Whereas the first possibility is supported by the observation that application of Aβ decreased the incorporation of Glut3 into the plasma membrane of cultured neurons [405], other data support the second possibility [151]. Gu and coworkers observed that the activation of calpain I in neurons was correlated with a decrease of GLUT3 protein, and provided evidence suggesting that this effect is due to calpain I-mediated proteolysis of GLUT3 at the N-terminus [151]. Since calpain I in neurons can be activated during over-stimulation of amino acid receptors [418], GLUT3/Glut3 may be downregulated during excitatory stress and promote AD emergence. Promotion or aggravation of AD in response to downregulation of glucose transporters may be explained by effects of intracellular glucose on O-AcNAcylation of Aβ and tau peptides [466]. Downregulation of GLUT1/Glut1 and GLUT3/Glut3 in endothelial cells and neurons leading to a decrease of intracellular d-glucose may cause a slow-down of the hexosamine biosynthetic pathway (HBSP) that is involved in the synthesis of uridine 5′-diphosphate-N-acetylglucosamine (UDP-GlcNAc) (Fig. 7). Since UDP-GlcNAc is the donor molecule for the transfer of *N*-acetylglucosamine (GlcNAc) to proteins catalyzed by O-GlcNAc transferase (OGT), GlcNAc modification of intraneuronal proteins may be reduced (Fig. 7). The reverse reaction is catalyzed by glycoside hydrolase O-GlcNAcase (OGA). O-GlcNAcylation of tau protein and γ-secretase which are involved in generation of Aß, was observed, and it was shown that neurotoxicity of tau and Aβ in animal AD models was reduced when OGA was inhibited [462, 466]. O-GlcNAcylation of tau protein decreases the hyperphosphorylation of tau that leads to the formation of neurotoxic tau oligomers [216, 234, 466]. Degradation of APP by the amyloidogenic pathway leading to the generation of neurotoxic Aβ was shown to be decreased when O-GlcNAcylation of APP in neurons was stimulated by inhibition of OGA [116, 187]. This effect may be due to stimulation of O-GlcNAcylation of APP and/or of γ-secretase [187, 205]. γ-Secretase is involved in APP degradation and is activated by O-GlcNAcylation. Taken together, the data indicate that GLUT1/Glut1 and GLUT3/Glut3 are related to AD emergence and/or progression.

## GLUT1 deficiency syndrome

### GLUT1 deficiency syndrome in humans

Rarely occurring neurological disorders based on decreased expression and/or function of GLUT1 in brain are subsumed as GLUT1 deficiency syndrome (GLUT1-DS) [28, 86, 90, 319]. In most cases, GLUT1-DS is caused by heterozygous single-nucleotide variants (SNVs) in the *SLC2A1* gene that provoke complete or severe impairment of functionality and/or expression of GLUT1 in brain [367]. The identified SNVs induce amino acid exchanges, exon deletions, frame shifts, and effects on regulation of transcription or translation [163, 225, 238, 443]. Recessive inheritance was assigned to SNVs leading to moderate impairment of GLUT1 functionality [208, 353]. SNVs in *SLC2A1* that induce major impairment of GLUT1 functionality or expression are lethal in homozygote carriers as indicated by animal models of GLUT1-DS [303, 431]. Carriers of SNVs in *SLC2A1* may develop different neurological symptoms dependent on the residual functionality of GLUT1 in brain capillaries and astrocytes in combination with differential genetic predisposition of the affected individuals [45, 86]. Being expressed in capillary endothelial cells and astrocytes, GLUT1 is pivotal for glucose uptake across the BBB and for the glycolysis in astrocytes providing glucose and L-lactate for neurons. The observed neurological disorders in GLUT1-DS represent different clinical manifestations of intellectual impairment, acquired microcephaly, epilepsy, and movement disorders [45, 86]. GLUT1-DS was first described as an early onset childhood epileptic encephalopathy [90]. With the description of additional cases associated with mutations in GLUT1, the phenotype spectrum was expanded by epileptic encephalopathy with different types of seizures, movement disorders, and paroxysmal events of non-epileptic origin [45, 86]. The observed epileptic seizures comprise subtle myoclonic limb jerking with alternating staring and eye-rolling, unresponsiveness, head bobbing, and generalized seizures. The movement disorders include ataxia, spasticity, and dystonia that occur in different combinations. The observed paroxysmal events comprise intermittent ataxia, periodic confusion, periodic weakness, and recurrent headaches. Additionally, specific atypical manifestations of GLUT1-DS have been described [45, 86]. They include paroxysmal exertion induced dystonia without and with seizures, intermittent ataxia, dystonia, migraine or choreoathetosis, and alternating hemiplegia.

The diagnosis of GLUT1-DS should be made in children as early as possible trying to prevent serious disease progression. A decreased d-glucose concentration in CSF called hypoglycorrhachia is a distinct biomarker of GLUT1-DS; however, it also shows up in some other neurological diseases [86, 226]. A second diagnostic marker is a decreased uptake of 2DOG or 3OMG into erythrocytes. This marker may not show up when GLUT1 expression is selectively downregulated in brain. Downregulation of GLUT1-mediated d-glucose uptake into brain can be detected by PET using [^18^F]DOG [2, 318]. For ultimate validation of GLUT1-DS, DNA sequencing of the *SLC2A1* gene should be employed. It is recommended to include non-coding gene regions to allow detection of mutations in regulatory domains [238, 443].

For therapy of GLUT1-DS, ketogenic diets (high-fat, low protein, low-carbohydrate) have been introduced trying to compensate impaired cerebral energy supply with glucose by short-chain fatty acids [90, 206, 207]. It was observed that the ketogenic diets improved various but not all symptoms of GLUT1-DS. If a ketogenic diet is started very early in life when brain development has not been completed, it may prevent the development of encephalopathy and alleviate the severity of the disease including intellectual deficits [81, 85]. Because compliance of ketogenic diets is bad in some patients, a modified Atkins diets (high-fat, high protein, low-carbohydrate) have been introduced [8]. Modified Atkins diets showed the same positive effects as the ketogenic diets.

### Animal models for GLUT1 deficiency syndrome

To establish animal models of GLUT1-DS, heterozygous Glut1 knockout mice [303, 431] and transgenic mice expressing antisense-Glut1 [166, 257] were generated. Homozygous Glut1 knockout mice proved to be lethal [303, 431] and antisense-Glut1 mice in which the expression of Glut1 was strongly suppressed showed very severe phenotypes of GLUT1-DS including anencephaly and pronounced cerebral dysgenesis [166]. The heterozygous Glut1 knockout mouse generated by Wang and coworkers exhibited a less severe GLUT1-DS phenotype compared to an antisense-Glut1 mouse described by Marin-Valencia and coworkers [257, 396, 408, 431].

In the heterozygous Glut1 knockout mouse with mild GLUT1-DS phenotype, no epileptic seizures and no distinct neuronal failures were observed. However, the mice exhibited impaired motor performance, motoric coordination, and learning [431]. In electroencephalograms, spontaneous generalized epileptiform discharges without behavioral correlates were observed [431]. Starting at the age of 21 weeks, the brain weight of the heterozygous Glut1 knockout mice was slightly smaller compared to wildtype mice [408, 431]. The plasma membrane abundance of Glut1 protein in brain was decreased by about 30% and the CSF-to-blood glucose ratio was decreased by about 70% [431]. [^18^F]DOG PET measurements indicated a decrease in cerebral glucose uptake. In these heterologous Glut1 knockout mice, also a slight decrease of the hippocampal volume and an increase of activated astrocytes in deeper cortical layers were observed [408]. Noteworthy, an expansion of small blood vessels in thalamus during brain development that was observed between 2 and 20 weeks after birth in wildtype mice, was significantly reduced in the heterozygous Glut1 knockout mice [396].

The transgenic Glut1-antisense mice with severe GLUT1-DS phenotype displayed severe ataxia [257]. These mice manifested generalized jerks during rest and motion and showed exaggerated response to tactile and acoustic stimuli. Different to wildtype mice, frequent epileptic spikes and series of spike and spike-wave activities were observed. The brain weight of the Glut1-antisense mice was reduced by about 8% and the concentration of Glut1 in forebrain was decreased by about 50%. Uptake of intraperitoneal injected [^14^C]2DOG into cerebral cortex and thalamus was decreased by about 30%. In brain tissue of the Glut-antisense mice, the abundance of acetyl-CoA and fatty acids were reduced whereas the concentrations of tricarboxylic acid cycle intermediates and of amine neurotransmitters were not changed. This suggests that the tricarboxylic acid cycle is intact and can be maintained by ketone body utilization. Oxidative phosphorylation appears to be sufficient to provide energy for neurotransmitter synthesis. The findings suggest that GLUT1-DS—at least in this model—was not due to an energy deficit. Accordingly, the hypothesis was raised that GLUT1-DS is caused by a shortage of acetyl-CoA that leads to downregulation of acetyl-CoA-dependent metabolic pathways such as the synthesis of fatty acids and lipids [257, 317]. Therapy with ketonic diets may prevent the shortage of acetyl-CoA.

## Stroke

### Pathophysiology and animal models

Stroke is a devastating neurological disturbance that is the second leading cause of death worldwide. More than 80% of stroke events are ischemic and result from restricted blood flow to a brain part. Stroke is mostly caused by arterial occlusion due to thrombosis, embolism, and/or arteriosclerosis. The arterial occlusion causes irreversible structural damages in a core region and changes in a surrounding area called penumbra that may be reversible [17, 237, 398]. Ischemia leads to failure of supply with d-glucose and oxygen that are required for ATP formation. In brain, ATP is mainly used to fuel the Na^+^-K^+^-ATPase and the Ca^2+^-ATPase in neurons that are pivotal for maintenance of transmembrane gradients of Na^+^, K^+^, and Ca^2+^ and of plasma membrane potential. Failure of ATP supply leads to an accumulation of intracellular Na^+^ followed by influx of monovalent anions such as Cl^−^ and influx of water, resulting in cytotoxic edema. The depolarization of plasma membranes induces opening of voltage-gated cation channels and reverses transport directions of the Na^+^/Ca^2+^ exchanger. The resulting increase of intracellular Ca^2+^ in neurons induces fusion of neurotransmitter containing vesicles with presynaptic membranes. Massive release of glutamate is neurotoxic and exacerbates neuronal damage by overstimulation of excitatory receptors. Energy failure in brain cells also promotes generation of reactive oxygen species (ROS) by mitochondria. ROS induce activation of inositol trisphosphate and ryanodine receptors liberating Ca^2+^ from intracellular stores. The massive cytosolic Ca^2+^ overload induced by these processes activates Ca^2+^-dependent proteases, phospholipases, endonucleases, and Ca^2+^-calmodulin-dependent nitric oxide synthases. The activation of these and other enzymes promotes protein degradation, DNA damage, and disruption of cellular signaling pathway leading to cellular death. Necrotic cells release cytotoxic compounds that may enter adjacent neurons with impaired plasma membrane integrity. Cerebral ischemia also induces inflammatory reactions. In addition, ischemia is associated with an increase of passive permeability of microvessels combined with changes of transporter abundance in capillary endothelial cells [398]. For example, it has been described that the activity of the Na^+^-K^+^-2Cl^−^-cotransporter in the luminal membrane of capillary endothelial cells was increased during ischemia [300]. These changes in the BBB cause vasogenic brain edema that represents a frequent cause of early mortality during stroke. Trying to improve the outcome of stroke events, an early recanalization of occluded vessels is attempted, e.g., by thrombolytic therapies or mechanical interventions [398]. These therapies may be beneficial promoting salvage of tissue in the penumbra region; however, they may also lead to an increase of the infarcted tissue volume [298]. Reasoning about the role of glucose transporters during stroke, the regulation of glucose transporters in the core region during the onset of cellular death and the regulation of glucose transporters in the penumbra must be considered. This includes regulation in the penumbra during later phases of stroke when ischemia may be blunted due to opening of collateral circulation and/or therapeutic recanalization.

The knowledge about regulation of glucose transporters in brain during stroke is derived from occlusion-reperfusion models in rodents. In the most frequently applied median cerebral artery occlusion (MCAO) model [223], the median cerebral artery (MCA) is occluded whereas in the bilateral common carotic artery occlusion (BCCAO) models, both common carotic arteries are clamped without or with parallel reduction of blood pressure [136, 265]. The artery occlusions were performed for short time periods of 6 to 15 min or for several hours. At different times after canceling the occlusion, the expression and/or function of glucose transporters was investigated. Different effects showed up at different times. Effects observed within hours are supposed to represent the early response to ischemia and direct counterregulations. At variance, the effects observed one or several days after occlusion represent long-lasting regulatory responses.

### Expression of glucose transporters during stroke

#### GLUT1

One hour after short-term MCAO in rats, Glut1 mRNA was upregulated throughout ipsilateral and contralateral brain cortex [223]. In the following hours, upregulation of Glut1 mRNA was normalized in contralateral cortex whereas it was intensified in a lateral region of the ipsilateral cortex. Upregulation of Glut1 was observed in microvessels, astrocytes, and distinct neuronal populations. One day after MCAO, Glut1 mRNA was still upregulated in glial cells of the penumbra but not anymore in neurons [223]. In another study on rats in which the MCA was occluded for 3 h, mRNA and protein of Glut1 were upregulated at 12 h of reperfusion in an ipsilateral cortical area outside the core infarct region [412]. BCCAO models of stroke were studied in gerbils and rats. Three hours after 6-min BCCAO in gerbils, Glut1 mRNA was increased in brain cortex and thalamus [136]. After 1 day, Glut1 mRNA in these regions was further increased whereas it was gone after 3 days. Employing BCCAO in combination with blood pressure reduction in rats, effects on Glut1 associated immunoreactivity were investigated in hippocampus [265]. One and 4 days after 15-min BCCAO, Glut1 protein in small blood vessels and in hippocampal tissue was upregulated. In parallel, total length and ramification of microvessels were increased. A study in rats with STZ-induced diabetes revealed that upregulation of Glut1 mRNA and protein in response to MCAO was more pronounced compared to non-diabetic rats [464].

**Fig. 8:**
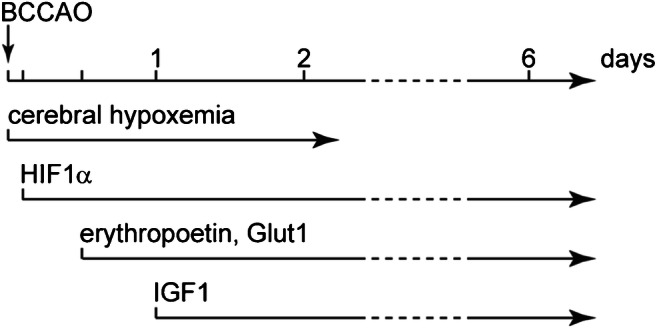
Chronical order of onset and duration of cerebral hypoxemia, upregulation of transcription factor HIFα, the HIF1α target proteins erythropoetin and Glut1, and IGF1 after stroke. The scheme is based on data in rats employing a BCCAO model of stroke [61]

Some information about mechanisms that may be involved in regulation of GLUT1/Glut1 during stroke is available. Thus, data were reported suggesting that heat shock protein (HSP) 70, hypoxia inducible factor (HIF) 1, and insulin-like growth factor (IGF) 1 are involved [61, 265]. One day after 15-min BCCAO in rats, upregulation of Glut1 protein in hippocampus was correlated with upregulation of HSP70 [265]. In another study in rat depicted in Fig. 8, 1 h after 12-min BCCAO, the expression of HIF1α was increased whereas expression of the HIF1α target proteins erythropoietin and Glut1 were increased only after 12 h [61]. Interestingly, upregulation of HIF1α, erythropoietin, and Glut1 persisted for 1 week although cerebral hypoxemia was only detectable for 2 days. Noteworthy, cerebral expression of insulin growth factor (IGF) 1 that stimulates the expression of HIF1α was increased only 1 day after ischemia but persisted for 1 week like HIF1α, erythropoietin, and Glut1. Based on these data, the hypothesis was raised that hypoxia in brain induces expression of HIF1α that stimulates the expression of various gene products including Glut1. Thereby a delayed and continuous upregulation of IGF1 may be promoted that drives the sustained expression HIF1α and the upregulation of Glut1. Data that were obtained with cultivated rat astrocytes suggest that NF-κB is involved in ischemic regulation of Glut1 in astrocytes [185]. It was observed that the upregulation of Glut1 in cultivated astrocytes in response to glucose and oxygen deprivation was blunted when NF-κB was inhibited.

#### GLUT3

The regulation of Glut3 during stroke differs to Glut1. For example, throughout the first couple of hours after short-term MCAO in rats, no changes of Glut3 mRNA were observed in the ipsilateral or contralateral forebrain at variance to Glut1 [223]. However, 1 day after MCAO when the increased expression of Glut1 mRNA was subsided, the abundance of Glut3 mRNA in neurons of the ipsilateral cortex was slightly increased. In another MCAO study in rats, it was observed that the expression of Glut3 mRNA and protein in the contralateral cortex were increased 2 days after 3-h MCAO [412]. Performing 6-min BCCAO in gerbils, similar results were obtained as after short-term MCAO in rats [136, 223]. Three hours after BCCAO, the abundance of Glut3 mRNA in cerebral cortex and thalamus was not changed at variance to Glut1 whereas after 1 day, Glut3 mRNA was increased in both regions. Different effects on Glut3 expression were observed in hippocampus of rats when a more severe ischemia was induced by 15-min BCCAO combined with blood pressure reduction [264]. In these experiments, a decrease of Glut3 protein was detected 4 days after BCCAO which became maximal after 7 days [264]. Performing MCAO in rats with STZ-induced diabetes, data were obtained indicating that the regulation of Glut3 in brain was influenced by homeostasis of blood glucose similar to Glut1 [464]. After MCAO in rats with STZ-induced diabetes, ipsilateral upregulation of Glut3 was more pronounced compared to non-diabetic rats [464].

Experiments on cultivated neurons and astrocytes provided information about the regulation of Glut3 in response to energy depletion during ischemia. Thus, energy depletion experiments on primary cerebellar granule neurons (CGNs) suggested that Glut3 is rapidly inserted into the plasma membranes during onset of ischemia [436]. This rapid posttranslational regulation may counteract neuronal cell death in response to ischemia. In these experiments, energy depletion was induced by excitation of CGNs with glutamate promoting cell death. Upon glutamate excitation the mitochondrial membrane potential was increased, intracellular ATP was decreased, AMP was increased, and AMPK was activated. Evidence was provided that the activation of AMPK promoted the recruitment of Glut3 to the plasma membrane leading to a protection of the CGNs from cell death [434, 436]. Data obtained with cultivated rat astrocytes suggest that Glut3 is upregulated in astrocytes during ischemia [185]. After glucose and oxygen deprivation, the expression of Glut3 in cultivated astrocytes was increased on mRNA and protein level. This upregulation was blunted after inhibition of the transcription factor NF-κB.

#### SGLT1

Experiments with cultivated porcine brain cells and stroke models in mice were performed to elucidate the impact of SGLT1/Sglt1 expressed in brain on the devastating effects of stroke [423, 450]. It was observed that SGLT1 expressed in primary cultured endothelial cells derived from small vessels of bovine brain was stimulated under hypoxemic, hypoglycemic, and/or aglycemic conditions of cultivation [423]. During cultivation under control conditions with 5.5 mM d-glucose in the medium, phloretin inhibited glucose uptake was observed that was probably mediated by GLUT1, whereas no significant phlorizin inhibited glucose uptake was detected. When the cells were cultivated for 12 h under hypoxemic conditions in the absence of d-glucose, phloretin inhibited d-glucose uptake was increased fivefold. Under these conditions, also phlorizin inhibited d-glucose uptake showed up that was sodium dependent. The data suggest that SGLT1/Sglt1-mediated uptake across the BBM is increased during ischemia.

To determine whether SGLT1/Sglt1 or another phlorizin inhibited member of the *SLC5* transporter family expressed in brain influences the outcome of stroke, MCAO experiments were performed in mice [423, 450–453]. In one experimental setting, the MCA was occluded for 6 h combined with i.p. injection of phlorizin or saline [423]. It turned out that phlorizin decreased the infarct volume, reduced brain edema, and blunted the decrease of deficit scores. In subsequent studies of Yamazaki and coworkers trying to elucidate the cause for the protective effect of phlorizin during stroke, two observations were considered [450–453]. First, the finding that blood glucose is increased during the early phase of brain ischemia [158, 261] and second, the observation that ischemia-induced tissue deterioration was protected by insulin [158]. In the studies by Yamazaki and coworkers, the MCA was occluded for 2 h and phlorizin was either subsequently applied by i.p. injection or phlorizin, d-glucose, and/or antisense SGLT1 RNA was(were) applied by i.c.v. injection. Fasting blood glucose, infarct size, deficit scores, and/or Sglt1 protein was(were) measured after 1 or 3 days. In the first study of this series, it was investigated whether phlorizin inhibited (a) *SLC5* transporter(s) that is(are) involved in generation of ischemic hyperglycemia and/or in d-glucose-dependent aggravation of ischemia-induced defects [450]. One day after MCAO, a decrease of fasting blood glucose was observed after i.p. but not after i.c.v. application of phlorizin. This indicates that cerebral *SLC5* type transporters were not critically involved in the generation of ischemic hyperglycemia. Of note, i.c.v. application of phlorizin after MCAO decreased the infarct size and improved deficit scores that were determined 3 days after MCAO. In addition, it was observed that infarct size and deficit scores were increased after i.c.v. injection of d-glucose or AMG and that these effects were blunted upon coinjection of phlorizin [450, 451, 453]. These data indicate that d-glucose interaction with a phlorizin inhibitable member of *SLC5* family in brain exacerbated tissue damage after MCAO. It was also demonstrated that 1 day after MCAO, the expression of Sglt1 protein was increased in brain cortex and striatum but not in hippocampus and hypothalamus [450, 452]. This upregulation probably occurred in neurons where Sglt1 is predominantly expressed [330, 452]. Noteworthy, downregulation of Sglt1 expression in brain by i.c.v. injection of antisense Sglt1 mRNA decreased infarct size and blunted deficit scores 1 day after MCAO [453]. Collectively, the data suggest that phlorizin-sensitive glucose uptake by Na^+^-d-glucose cotransport in brain cells aggravate tissue destructions during brain ischemia. They implicate that sodium-mediated d-glucose transport via upregulated SGLT1/Sglt1 in neurons plays a critical role in this effect. In contrast to d-glucose transport via GLUT transporters, Na^+^-d-glucose transport is an energy-consuming process that may increase energy deficit during brain ischemia. In accordance with this hypothesis, it was demonstrated in primary cultured mouse neurons expressing Sglt1 that application of extracellular d-glucose increased intracellular Na^+^ and that the increase of Na^+^ could be inhibited by phlorizin [453].

### Effects of estrogen, ascorbic acid, and curcumin on glucose transporters during stroke

#### Estrogen

Estrogen replacement in postmenopausal women has been shown to be correlated with improved outcome of stroke [145]. This may be due to effects of estrogen on the Na^+^-K^+^-Cl^−^ cotransporter or on glucose transporters expressed in brain [301, 374]. It was observed that 17beta-estradiol increased the expression of Glut1 in the BBB of ovarectomized female rats [373] and the expression of GLUT1, GLUT3, and GLUT4 in brain of ovarectomized female rhesus monkeys [63]. In female ovarectomized rats, the effect of one subcutaneous injection of 17beta-estradiol on infarct size and Glut1 expression was investigated [374]. In these experiments, the MCA was occluded for 30 min and infarct size and cerebral Glut1 expression were investigated 1 day later. After 17beta-estradiol treatment, the infarct size was decreased by about 30% and the expression of Glut1 protein in the infarcted core region was reduced by about 20%. In the penumbra of the infarct, the expression of Glut1 protein was about 20% increased in response to 17beta-estradiol treatment [374].

#### Ascorbic acid

After food supplementation of non-diabetic and diabetic rats with ascorbic acid (AA), protective effects on infarct size, brain edema, and neurological deficits after MCAO were observed [186]. In the experiments, diabetes was induced by STZ and AA was provided for 2 weeks. Thereafter, MCAO was performed for 2 h, and infarct size, brain edema, neurological deficits, and expression of cerebral Glut1 were analyzed 1 day later. In brain of sham-operated diabetic rats, the expression of Glut1 was smaller compared to sham-operated non-diabetic rats. However, AA induced upregulation of Glut1 expression in diabetic and non-diabetic rats to similar levels. After MCAO without AA treatment, infarct size, brain edema, and neurological deficits were more pronounced in diabetic rats compared to non-diabetic animals. AA treatment improved the outcome after MCAO in non-diabetic and diabetic rats; however, the improvement in diabetic rats was more pronounced. The data suggest that the protective effect of AA on neuronal survival during ischemia is partially due to an AA-induced upregulation of Glut1 [70].

#### Curcumin

Curcumin extracted from turmerin is widely used as food additive. Curcumin exhibits various pharmacological effects including metabolic, anti-inflammatory, antioxidant, and antidiabetic effects [48, 59]. Recent data performed in rats with STZ-induced diabetes suggest that curcumin compensates the decreased expression level of GLUT1/Glut1 during diabetes and thereby improves the outcome of stroke [448]. In rats with STZ-induced diabetes, 90-min MCAO was performed and the animals were subsequently gavaged with curcumin or saline as control. One day later, neurological deficit scores, infarct volume, and brain edema were determined. After curcumin treatment, infarct size and brain edema were reduced and neurological deficit scores improved. In parallel, curcumin blunted the MCAO-induced decrease of cerebral expression of Glut1 and Glut3 protein. In tissue culture experiments, it was demonstrated that curcumin increased the expression of Glut1 and Glut3 on the cellular level.

## Traumatic brain injury

### Pathophysiology and animal models

TBI frequently causes mental and physical disabilities. The term TBI comprises focal and diffuse brain damage caused by different types of violation and brain concussion. For example, brain contusion may induce an instant focal damage followed by secondary focal and/or secondary diffuse brain damage whereas acceleration/deacceleration trauma may only cause a delayed diffuse damage [437]. The instant traumatic insult during brain contusion is followed by different stages of secondary damage as observed during stroke. Early stages of secondary damage during TBI are characterized by lack of oxygen and glucose supply that is caused by destruction and/or occlusion of brain vessels combined with impairment of cerebral blood flow [315, 461]. Failure of oxygen and glucose supply induces metabolic responses in neurons and astrocytes promoting anaerobic glucose metabolism that leads inter-alia to increased cerebral l-lactate concentrations and decreased ATP concentration in neurons [32, 88, 141, 413]. As described for stroke, lack of ATP induces a cascade of processes that include plasma membrane depolarization, changes in ion distribution, release of neurotransmitters, increased permeability of brain vessels, edema, and formation of ROS. Excitatory toxicity, ROS and brain edema may promote secondary brain damage [27]. In later stages during TBI, cell death in severely damaged tissue promotes immigration of immune cells [463]. The edema recedes and blood circulation may improve in less severe damaged tissue regions by an increase of capillary length and capillary diameter. In regions with less severe tissue damage, regulatory processes take place that include regulation of metabolic pathways and glucose transporters. Remarkably, TBI also induces extracerebral body responses that in return influence metabolic regulations in damaged brain tissue. One important body response to TBI is a post-traumatic hyperglycemia that is correlated with unfavorable clinical outcome [88, 221, 356].

The knowledge about pathophysiology of TBI is mainly derived from studies on animal models. Various animal models for TBI have been applied in rodents. The employed models include the fluid percussion injury (FPI) model [100, 461], the controlled cortical impact (CCI) model [101, 352], the impact acceleration model [168], and closed head injury (CHI) models [410]. These models mimic brain contusion of different severity and diffuse brain trauma without contusion.

During TBI, changes in cerebral d-glucose uptake and cerebral expression of the glucose transporters Glut1, Glut3, and Sglt1 have been described. Whereas in areas of badly damaged tissue entering cell death, transporter abundance is decreased like other cell proteins, specific, time-dependent regulatory processes have been observed in less severely damaged areas.

### Cerebral d-glucose uptake and cerebral expression of glucose transporters during TBI

#### Cerebral d-glucose uptake

After brain trauma, changes in cerebral d-glucose uptake, cerebral glucose metabolism, and cerebral expression of glucose transporters were observed. In patients, cerebral uptake of d-glucose was decreased during the acute phase of TBI indicating hypometabolism [80]. Employing [^18^F]DOG PET, it was observed that cerebral glucose uptake in patients was increased 1 week after severe brain trauma [32]. In rats, local cerebral metabolic rates for 2DOG were studied in FPI models for TBI [202, 457]. After mild unilateral FPI mimicking brain concussion, the local cerebral metabolic rate for 2DOG was increased for 30 min in cerebral cortex and hippocampus [202, 457]. This increase of 2DOG utilization was blunted by cerebral application of glutamate receptor antagonists. Between 6 h and 5 days after the FPI, the 2DOG utilization was decreased [457]. In another study in rats in which more severe FPI was performed, the local cerebral metabolic rate for 2DOG was decreased for 1 day [95].

#### GLUT1 and GLUT3

Immunoreactivity of an antibody against the human erythroid 55 kDa GLUT1 transporter was investigated by light and electron microscopy in brain cortex that had been resected from patients about 8 h after brain trauma [76]. At this time, a decrease of immunoreactive small blood vessels was observed close to the damaged area whereas the numbers of immunoreactive small blood vessels were increased in areas located more distantly. Blood vessels in these more distant areas exhibited a more intense immunoreactivity than blood vessels close to the damaged area suggesting an upregulation of GLUT1 expression. Immunoelectron microscopy revealed that the GLUT1-related immunoreactivity was predominantly located in endothelial cells of brain capillaries.

In brains of rodents, effects of TBI on immunoreactivity of 55 kDa Glut1 polypeptide expressed in brain capillaries and of 45 kDa Glut1 polypeptide expressed in glial cell was investigated employing a severe impact acceleration model and a CHI model [157, 366, 410]. In both models performed in rat, in which severe diffuse brain injury was induced, no effects on the expression of the 45 KDa Glut1 polypeptide were observed up to 2 days after the traumatic events [157, 410]. In contrast, the expression of the 55 kDa Glut1 protein was increased 6 h and 2 days after the trauma [410]. Employing a CCI model in mice, it was observed that mRNA of Glut1 was not changed 1 day after the trauma [366]. Together, the data suggest that GLUT1/Glut1 in the BBB is post-transcriptionally upregulated after TBI.

In rodents, data were obtained which suggest that HIF1α is involved in regulation of Glut1 expression after TBI. After TBI, HIF1α was increased in parallel with 55 kDa Glut1 polypeptide, aquaporins and other proteins whereas the expression of 45 kDa Glut1 protein was not changed [98, 99, 175, 410]. Inhibition of HIF1α by acriflavine increased the cerebral expression of 55 kDa Glut1 polypeptide in control mice and altered its regulation after TBI [410]. At variance, acriflavine did neither influence the expression of 45 kDa GLUT1 polypeptide in control mice nor in mice after TBI [410]. After inhibition of HIF1α, the expression of the 55 kDa Glut1 polypeptide was decreased 6 h after severe CHI. HIF1α was shown to be involved in the protective effect of heat acclimation (HA) during TBI. HA denotes a prolonged exposure of an animal or human individual to a moderately high ambient temperature. In rodent models, it was observed that HA reduced tissue damage and cerebral impairment during TBI [370, 409, 410]. HA increased the cerebral expression of HIF1α and of 55 KDa Glut1 polypeptide in control mice whereas it did not alter the expression of the 45 kDa Glut1 polypeptide. After HA, upregulation of the 55 kDa Glut1 polypeptide after TBI was maintained, however, no neuroprotective effect of HA during TBI was observed when HIF1α was inhibited [410]. At variance, upregulation of the 45 kDa Glut1 polypeptide during TBI was only observed after HA and this upregulation was prevented when HIF1α was inhibited.

After TBI in rats induced by an impact acceleration model, a distinct upregulation of Glut3 protein was observed in cerebral cortex and cerebellum 4 h after TBI [157]. Two days after TBI, upregulation of GLUT3 was still detectable.

#### SGLT1

Concerning expression and function of SGLT1/Sglt1 in brain during TBI, only very limited information is available. In one study, SGLT1-related immunoreactivity in Western blots was compared between cerebral tissues from dissected human bodies that died following TBI and due to cardiac arrest [302]. In female and male individuals that died after TBI, higher expression of SGLT1 was observed compared to the cardiac arrest group.

Employing a CCI model in wildtype mice and in mice in which the regulatory protein Rs1 (*Rsc1A1*) was removed [310], expression of Sglt1 mRNA, infarct size, brain edema, and motoric disability were compared [366]. Removal of Rs1 had no effects on the expression of Sglt1 mRNA in cerebral cortex and hippocampus; however, it altered the regulation of Sglt1 after TBI. Whereas in brain cortex of wildtype mice Sglt1 mRNA was increased about 2.5-fold 1 day after TBI, no increase of Sglt1 mRNA was observed in Rs1 knockout mice. Importantly, in Rs1 knockout mice, infarct size, brain edema, and motoric disability were smaller than in wildtype mice whereas the posttraumatic increase of the cerebral d-glucose concentration was not changed. The data suggest that upregulation of SGLT1/Sglt1 during TBI aggravates secondary tissue damage and clinical outcome. The hypothesis was raised that upregulation of SGLT1/Sglt1 mediated Na^+^-d-glucose cotransport into neurons leads to an increased energy consumption that enhances tissue damage.

## Conclusions

The reviewed data show pivotal involvements of cerebral glucose transporters in various physiological brain functions and pathophysiological mechanisms associated with brain diseases. Despite extensive research during the last 40 years, most functions of glucose transporters in brain are poorly understood. This is due to the high complexity of brain functions involving glucose transporters and to technical difficulties to analyze mechanisms that are involved in specific brain functions. Other reasons are the overlap in substrate specificities and cerebral locations of glucose transporters and the complex regulations of glucose transporters in response to physiological and pathophysiological conditions. In addition, analysis of transporter locations in brain is complicated due to methodological limitations in the immunohistological analysis of transporter locations. The large majority of immunohistochemical localizations of glucose transporters in brain and most in vivo investigations have been performed on rodents and rodent models for diseases. Thus, most of our present knowledge concerns the functions of glucose transporters in rodents and does not necessarily reflect the situation in humans. Considering the high functional importance of glucose transporters in brain and their high biomedical impact intensive future research is demanded. This should include a detailed immunohistochemical localizations of the different glucose transporters in human tissue and a comparison of PET measurements using glucose analogs with different substrate specificities for glucose transporters between human individuals and rodents. In addition, further sophisticated in vivo experiments in rodents employing targeted knockout of selective glucose transporters in brain are required. An advanced understanding of the physiological and pathophysiological roles of glucose transporters in human brain will open the possibility to develop drugs that target cerebral glucose transporters. Such drugs may be useful for treatment of neurological disorders that are combined with cerebral energy deficiency such as stroke, TBI, and AD.

## Abbreviations

AA: Ascorbic acid
AD: Alzheimer’s disease
AβP: Amyloid beta-peptide
AMG: α-Methyl-d-glucoside
AMPK: AMP-activated protein kinase
APP: Amyloid precursor protein
ARH: Hypothalamic arcuate nucleus
BBB: Blood-brain barrier
BCCAO: Bilateral common carotic artery occlusion
CCI: Controlled cortical impact
CHI: Closed head injury
CGN: Cerebellar granule neuron
2DOG: 2-Deoxy-d-glucose
DIO: Diet-induced obesity
DMH: Dorsomedial hypothalamus
EGP: Endogenous glucose production
ER: Endoplasmic reticulum
FDOG: 2-Fluoro-2-deoxy-d-glucose
FDOG-6-P: FDOG phosphorylated in position 6
FPI: Fluid percussion injury
GABA: γ-Aminobutyric acid
GE: d-Glucose-exitated
GI: d-Glucose-inhibited
GK: Glucokinase
GlcNAc: *N*-Acetylglucosamine
GLUT1-DS: GLUT1 deficiency syndrome
HA: Heat acclimation
HBSP: Hexosamine biosynthetic pathway
HFD: High-fat diet
HIF: Heat inducible factor
HSP: Heat shock protein
i.c.v.: Intracerebroventricular
IGF: Insulin growth factor
IGTT: Intraperitoneal glucose tolerance test
ITT: Insulin tolerance test
K2P: Two-pore-domain potassium
LHA: Lateral hypothalamic area
MCA: Medial cerebral artery
MCAO: Medial cerebral artery occlusion
ME: Median eminence
NFT: Neurofibrillar tangle
OGA: O-GlcNAcase
OGT: O-GlcNAc transferase
3OMD: 3-*O*-Methyl-d-glucose
PD: Parkinson’s disease
PET: Positron emission tomography
ROS: Reactive oxygen species
SA: Spontaneous alteration
SNV: Single-nucleotide variant
SP: Senile plaque
STZ: Streptozotocin
TBI: Traumatic brain injury
T1DM: Type 1 diabetes mellitus
T2DM: Type 2 diabetes mellitus
UDP-GlcNAc: uridine 5′-diphosphate-*N*-acetylglucosamine
VDCC: Voltage-dependent Ca^2+^ channel
VMH: Ventromedial hypothalamic nucleus
ZDF: Zucker diabetic fatty
